# Inactive-to-Active Transition of Human Thymidine Kinase 1 Revealed by Molecular Dynamics Simulations

**DOI:** 10.1021/acs.jcim.1c01157

**Published:** 2021-12-17

**Authors:** Samanta Makurat, Zoe Cournia, Janusz Rak

**Affiliations:** † Faculty of Chemistry, University of Gdańsk, Wita Stwosza 63, 80-308 Gdańsk, Poland; ‡ Biomedical Research Foundation, Academy of Athens, 4 Soranou Ephessiou, 11527 Athens, Greece

## Abstract

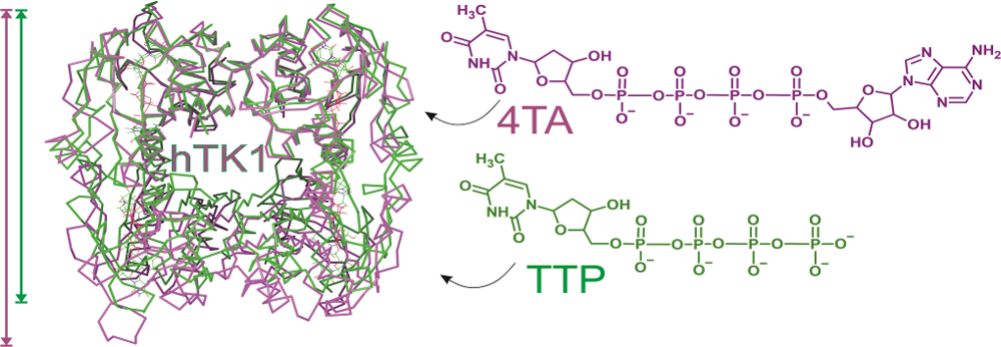

Despite its importance in the nucleoside (and nucleoside prodrug) metabolism, the structure of the active conformation of human thymidine kinase 1 (hTK1) remains elusive. We perform microsecond molecular dynamics simulations of the inactive enzyme form bound to a bisubstrate inhibitor that was shown experimentally to activate another TK1-like kinase, *Thermotoga maritima* TK (*Tm*TK). Our results are in excellent agreement with the experimental findings for the *Tm*TK closed-to-open state transition. We show that the inhibitor induces an increase of the enzyme radius of gyration due to the expansion on one of the dimer interfaces; the structural changes observed, including the active site pocket volume increase and the decrease in the monomer–monomer buried surface area and of the number of hydrogen bonds (as compared to the inactive enzyme control simulation), indicate that the catalytically competent (open) conformation of hTK1 can be assumed in the presence of an activating ligand.

## Introduction

**Figure 1:**
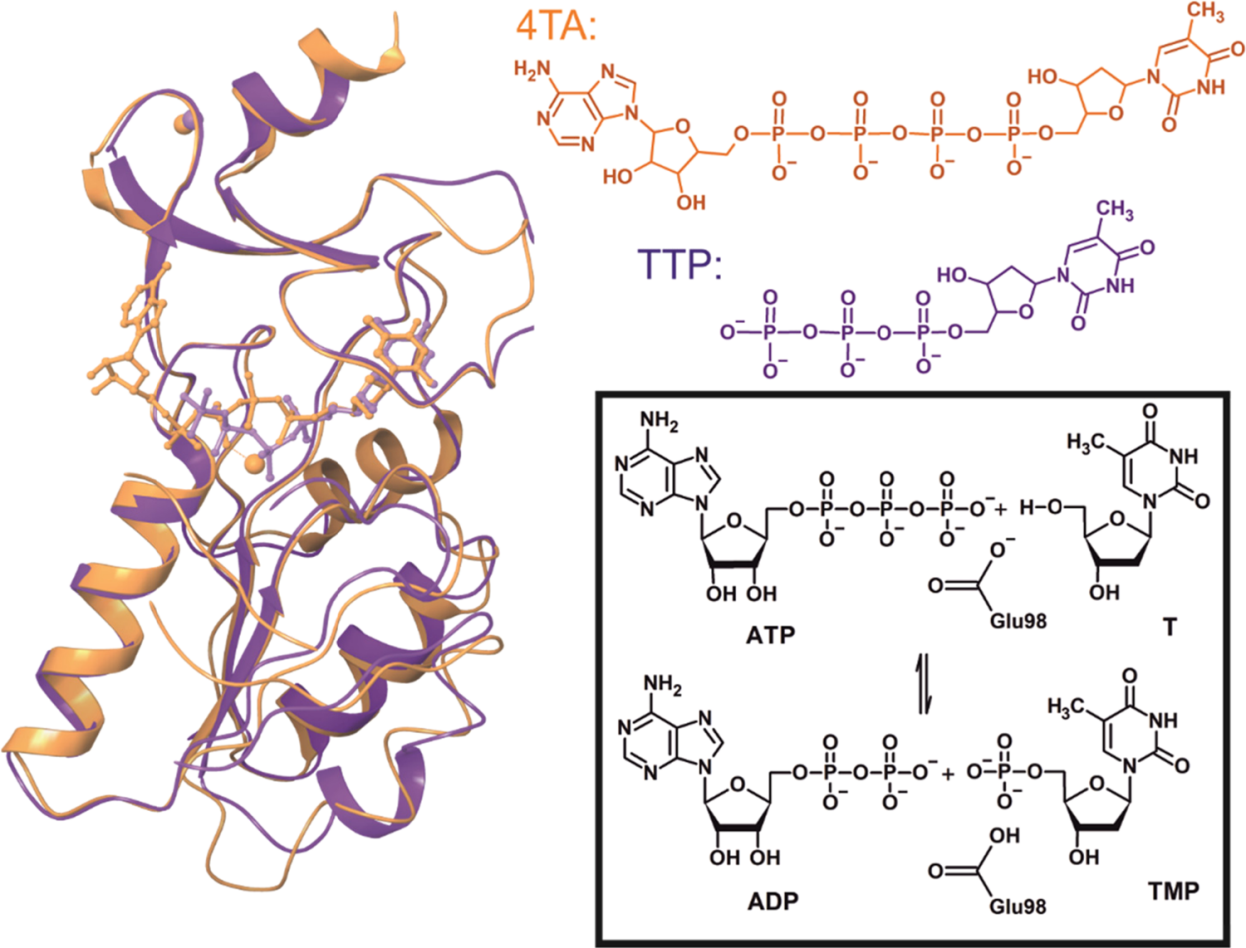
hTK1 (PDB code: 2ORV, violet) overlapped with *Tm*TK (PDB code: 2ORW, orange).^12^ Only one monomer is shown for each protein. hTK1 is cocrystallized with TTP and *Tm*TK with the bisubstrate inhibitor, 4TA. The phosphoryl transfer reaction catalyzed by hTK1 that leads to thymidine monophosphate (TMP) formation is shown in the frame. Glu98 serves as a base accepting the proton of the 5′-OH-group of T.

Human thymidine kinase 1 (hTK1) is a phosphotransferase that catalyzes phosphoryl transfer from adenine triphosphate (ATP) to thymidine (T) using glutamic acid, Glu98, as a proton acceptor (Figure 1).^1^ The enzyme, crucial for nucleoside metabolism related to DNA synthesis, is also clinically important for the activation of otherwise nontoxic prodrugs such as azidothymidine, cytarabine, acyclovir,^1,2^ and potentially radiosensitizing nucleosides that have to be incorporated into DNA prior to its radiation-induced damage. The latter nucleosides are being developed in our lab,^3,4^ and 5-iodo-4-thio-2′-deoxyuridine, 5-selenocyanato-2′-deoxyuridine, and 5-trifluoromethanesulfonyl-2′-deoxyuridine comprise the most recent ones.^5,6^

Despite being one of the essential enzymes in the nucleoside salvage pathway, hTK1 is also intriguing because of its different evolutionary origin compared to the remaining deoxyribonucleoside kinases.^7^ However, the mechanistic details of enzymatic reactions catalyzed by the protein have not been elucidated so far. The topology of TK1-like enzymes (structurally related TKs from other organisms) differs from that of other deoxynucleoside kinases. They form a dimer of dimers in their most active state,^8^ with two distinct protein–protein interfaces (referred to as weak and strong dimer interfaces) of which one is being stabilized by the protein–ligand interactions.^7,9^ The ATP-dependent dimer-to-tetramer transition is associated with 30-fold higher catalytic efficiency.^10^ The difficulty in describing the structural details of the mechanism of action of hTK1 mainly arises from the difficulty in crystallizing its active state in an open conformation. Thus far, the 3D structure of this enzyme in its active form has not been obtained.^11^ However, it has been suggested for another TK1-like enzyme from hyperthermophilic bacteria, *Thermotoga maritima* TK (*Tm*TK), for which the crystal structure of both inactive and active states is solved, that in the absence of a substrate in the phosphoryl donor-binding site the enzyme preferentially exists in a closed conformation. Upon ATP binding, the *Tm*TK enzyme significantly changes its quaternary structure leading to the catalytically competent (open) conformation.^11^ On the other hand, hTK1 is readily inhibited (and the enzyme conformation assumes then its closed form) by the feedback inhibitor, thymidine triphosphate (TTP), which binds to the phosphoryl acceptor-binding site.^12^

These traits, along with the fact that for hTK1-like enzymes the reaction occurs between the weak dimer interface (ATP adenosyl moiety in one monomer is stabilized by the amino acids of the neighboring monomer) and a flexible lasso loop on the other side (Figure 1),^12^ make the crystallographic structural investigations unusually difficult. Indeed, structural data available for hTK1 are limited. Namely, the Protein Data Bank (PDB)^13^ lists only four structures (PDB codes: 2WVJ,^14^ 2ORV,^12^ 1W4R,^9^ and 1XBT^7^), none being in the active conformation. Moreover, none of the deposited hTK1 structures has both native ligands bound (only the product of phosphorylation of T bound at the phosphoryl acceptor site was observed in X-ray experiments).^7,9,11−14^ Finally, only one of the PDB structures (1W4R) includes the full amino acid sequence of the active site.

Because of these inherent difficulties in obtaining structural data for hTK1, all investigations on the active structure and conformational changes of hTK1 are mostly based on the *Tm*TK crystal structure. *Tm*TK exhibits 36% identity and 55% similarity at the sequence level with hTK1 but, unlike hTK1, it crystallizes not only in the presence of TTP but also with the bisubstrate inhibitor P1-(5′-adenosyl)P4-(5′-(2′-deoxy-thymidyl))tetraphosphate (4TA, see Figure 1).^12^ The 4TA ligand, that occupies both sides of the enzymatic pocket, induces the active conformation of the protein. Unfortunately, crystallization of hTK1 with 4TA did not succeed, and only a complex with TTP in the inactive conformation of hTK1 was solved for the human enzyme. Such a conformation is not suitable for performing drug-design-related studies because the enzymatic pocket is closed and any attempts of docking ATP, which is the native ligand of hTK1 on the donor-binding site, fail. Access to a reliable active conformation of hTK1 is crucial for a hybrid, QM/MM approach leading to a sound description of thermodynamics and kinetics of the enzymatic phosphorylation.

In this study, we perform microsecond-long molecular dynamics (MD) simulations using available (inactive) hTK1 structures and the 4TA ligand (known to promote the active conformation in *Tm*TK) to computationally “activate” the hTK1 enzyme and observe the related conformational changes of the inactive-to-active transition.

## Results and Discussion

To cross-check and validate the reproducibility of the results and the simulation protocol, five different models were constructed and simulated in several replicas. Four control simulations (Table 1) were constructed as follows: (i) hTK1 complexed with TTP (hTK1-TTP) in the closed form, based on 1W4R^9^ with small refinements using the 1XBT^7^ structure, (ii) hTK1-TTP in the apo form having the ligand TTP removed from the structure (hTK1-apo), (iii) *Tm*TK complexed with 4TA (*Tm*TK-DIM) in the active form, the dimer of 2ORW,^12^ and (iv) the *Tm*TK-DIM duplicated to form a homotetramer (*Tm*TK-TET), which was created after observing high instability of the adenosine moiety in the *Tm*TK-DIM simulation, possibly due to the lack of a weak dimer interface that constitutes the adenosine-binding site. Finally, the simulation in which we expected to observe conformation opening was performed using the closed form of the hTK1 tetramer (as in hTK1-TTP), where the TTP ligand was replaced by the 4TA ligand (hTK1-4TA). Three independent copies starting from the same restart file after minimization, heating, and equilibration were run for most systems.

**Table 1:** List of Simulated Systems

name	protein	ligand	simulation time	remarks
hTK1-TTP	tetramer of chain A of 1W4R PDB structure	TTP	1 μs, three replicas	closed (inactive) conformation from crystal structure—control simulations
hTK1-apo	tetramer as in hTK1-TTP		700 ns, one replica	
*Tm*TK-DIM	dimer resembling 2ORW PDB structure	4TA	1 μs, three replicas	open (active) conformation from crystal structure—control simulations
*Tm*TK-TET	*Tm*TK-DIM duplicated to form a tetramer	4TA	1 μs, three replicas	
hTK1-4TA	tetramer as in hTK1-TTP	4TA	1.3 μs, three replicas	closed enzyme conformation bound to the inhibitor

**Figure 2:**
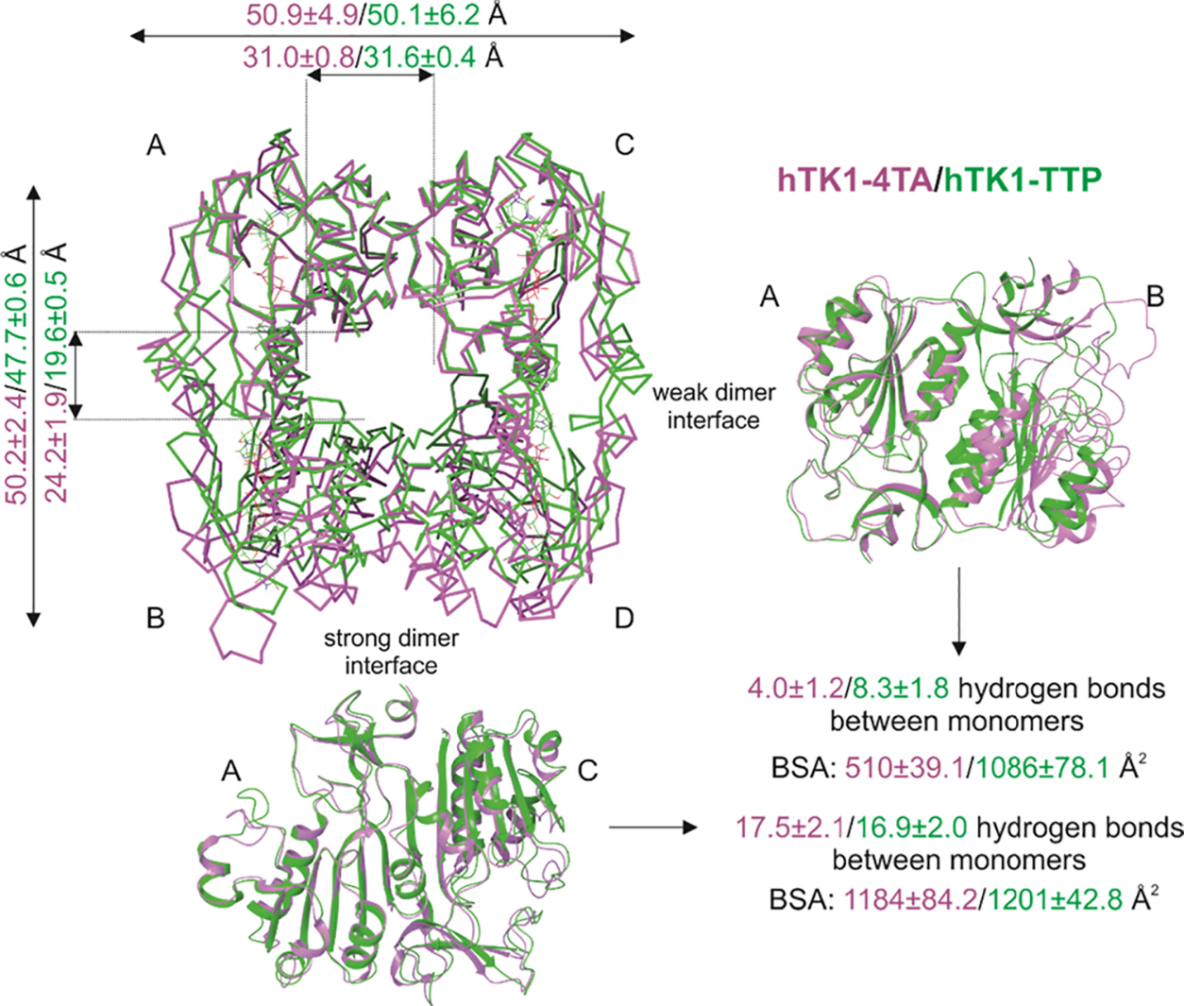
Overlap of hTK1-4TA (pink) and hTK1-TTP (green) most populated cluster representatives (first replica). In the top view of the tetramer, B and D monomers in the hTK1-4TA structure are visibly shifted down because of the expansion on the weak dimer interface. Additionally, the strong and weak dimer interfaces’ front views are shown. The corresponding distances between the monomers (as measured between chosen atom-pairs distances averaged over the last 900 ns of each replica) and hydrogen bonds (averaged over the last 900 ns of each replica) and the buried surface area (BSA) (averaged over all replicas most populated representatives) between monomers are shown in respective pink and green colors for hTK1-4TA and hTK1-TTP.

Conformational changes in TK-1-like enzymes induced by ATP involve the increase of the total volume of the tetrameric complex along the weak dimer interface,^11,12,15^ which is surmised to be critical for the proper orientation of ATP, as otherwise there is no space to fit the adenosine moiety. Indeed, the radii of gyration of all Cα atoms are on average ∼1 Å^3^ lower for hTK1-TTP and hTK1-apo simulations than for the hTK1-4TA simulations ones despite the same amino acid composition (Figure S1). Selected atom pair distances were evaluated for both hTK1 systems (reported in Figure 2, Figure S2, and Table S1) showing that on average the distances in the strong dimer interface differ between the compared complexes by no more than 1 Å during the whole simulation, while the dimension across the weak dimer interface expands by an average of 2.5 ± 2.4 to 4.6 ± 1.0 Å for hTK1-4TA (unlike for hTK1-TTP). This result is in excellent agreement with the crystal structures in closed and open states reported by Segura-Peña et al. for *Tm*TK. In these experimental studies, the distance across the weak dimer interface is reported to increase from ∼51 to ∼56 Å between the closed and open states^11^ and suggests that a transition to the open, catalytic state has been obtained in our simulations. This conformational change is also accompanied by the change of the binding pocket cavity volume (defined by the binding site amino acids from Welin et al.^7^), as measured with the EPOCK^16^ VMD^17^ plugin for all hTK1 trajectories. The average cavity volume varies from 680.1 ± 54.1 to 925.7 ± 194.5 Å^3^ for hTK1-4TA and from 405.6 ± 58.0 to 494.3 ± 76.3 Å^3^ for hTK1-TTP, depending on the chain and replica (see Table S2 and Figure S3) despite the same protein starting points for all simulations.

Transition to the open state is also indicated by the changes related to BSAs. The latter were calculated between the monomers of the most populated clusters with dr_sasa.^18^ Decreasing the BSA upon opening the conformation (from an average of 1086.9 ± 78.1 Å^2^ for hTK1-TTP clusters to an average of 510.6 ± 39.1 Å^2^ for the hTK1-4TA clusters) in the weak dimer interface and stable BSA for the strong dimer interface (1201.8 ± 42.8 and 1184.0 ± 84.2 Å^2^ for hTK1-TTP and hTK1-4TA, respectively) is consistent with the experimental values for *Tm*TK (Figure 2, Table S3; 370/1030 (weak dimer interface) and 1050/1030 Å^2^ (strong dimer interface) are reported for *Tm*TK open/closed states).^11^

**Figure 3:**
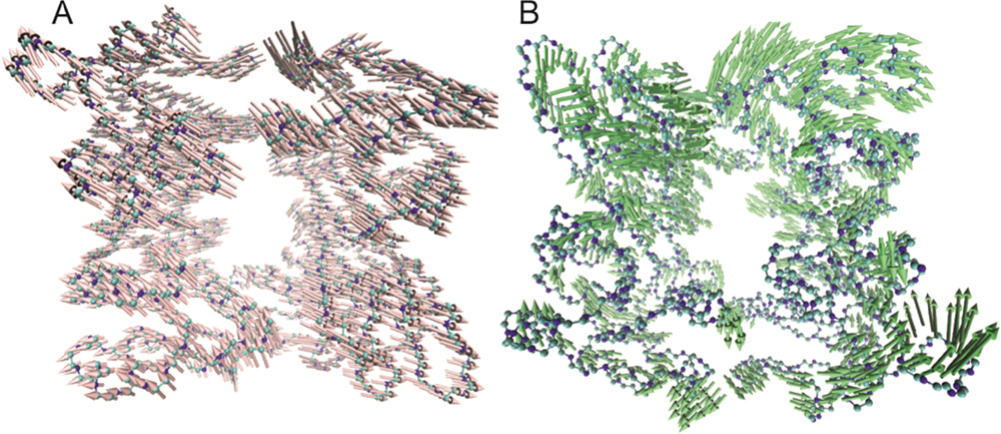
First (A) and second (B) PCs of hTK1 simulations are opening and twisting the tetrameric structure, as shown by the arrows.

**Figure 4:**
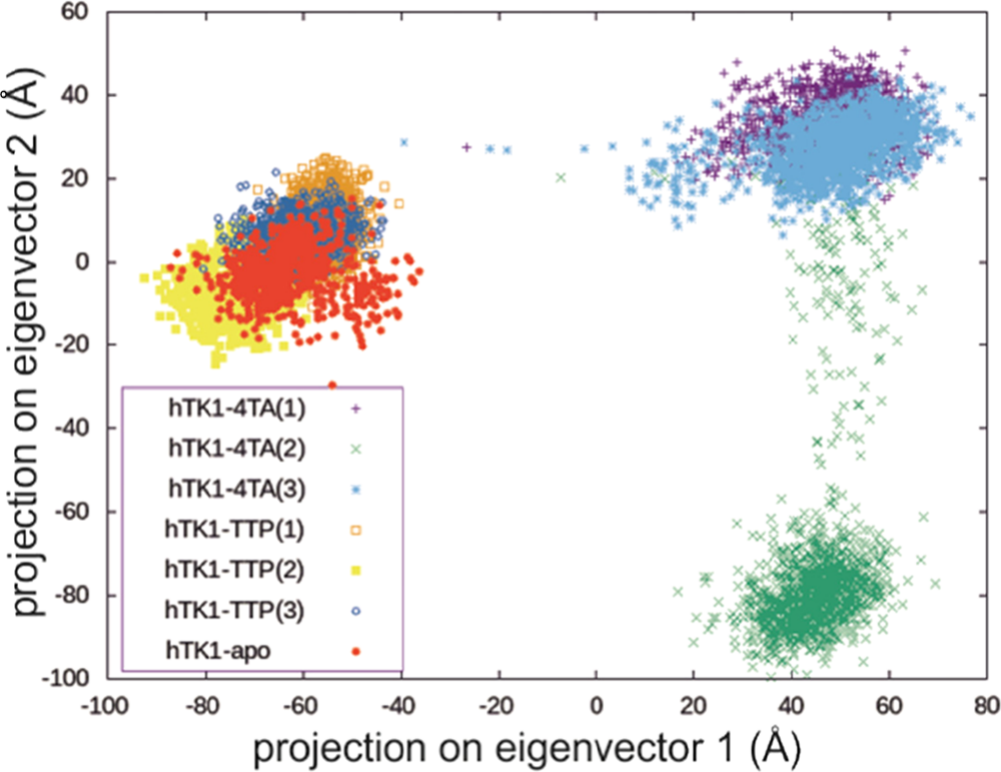
PC projection of the replica simulations shows a great overlay for hTK1-TTP and hTK1-apo structures and the expected closed state for hTK1-TTP and the open state for hTK1-4TA simulations.

Finally, principal component analysis (PCA) was performed for all hTK1 replica backbone atoms to identify the most important modes of motion.^19,20^ The PCA method also provides a metric to compare the conformational space of the different trajectories, as the protein motional modes can be represented by the first two principal components (PCs).^21^ The first two PCs correspond here to the opening and twisting of the hTK1 monomers, respectively, as shown in Figure 3. The PC projection calculated for the overlap of hTK1-TTP and hTK1-apo replicas occupies the same region (Figure 4), which shows that the closed and untwisted state of the tetramer remains stable during the simulations. For the hTK1-4TA replica simulations, more PC variance is shown. Indeed, while for hTK1-4TA all replicas are in the open state (depicted on the right of Figures 4 and 3A), the second replica of hTK1-4TA is additionally twisted compared to the other ones (see Figures 4 and 3B). This difference is in line with other observations (cf. Tables S1–S4, Figure S3). These may be related to the expected twist (the twist of ∼11°, which was previously described for the *Tm*TK structure in the literature^11^) or could potentially describe the loss of the weak monomer interaction that finally leads to the tetramer-to-dimer transition.

The number of hydrogen bonds between monomers on the weak dimer interface is reduced to half in the hTK1-4TA structure (4.0 ± 1.2 hydrogen bonds) with respect to the hTK1-TTP one (8.3 ± 1.8 hydrogen bonds), averaged over the whole simulation time and all replicas. For the strong dimer interface, the number of hydrogen bonds (∼17) does not depend on the ligand, 4TA or TTP (cf. Figure 2 and Table S4). Similarly, the control simulations for the active *Tm*TK-TET, bonded with the 4TA ligand, exhibit an average of 11.3 ± 2.4 hydrogen bonds on the strong dimer interface and 3.5 ± 1.3 on the weak one.

**Figure 5:**
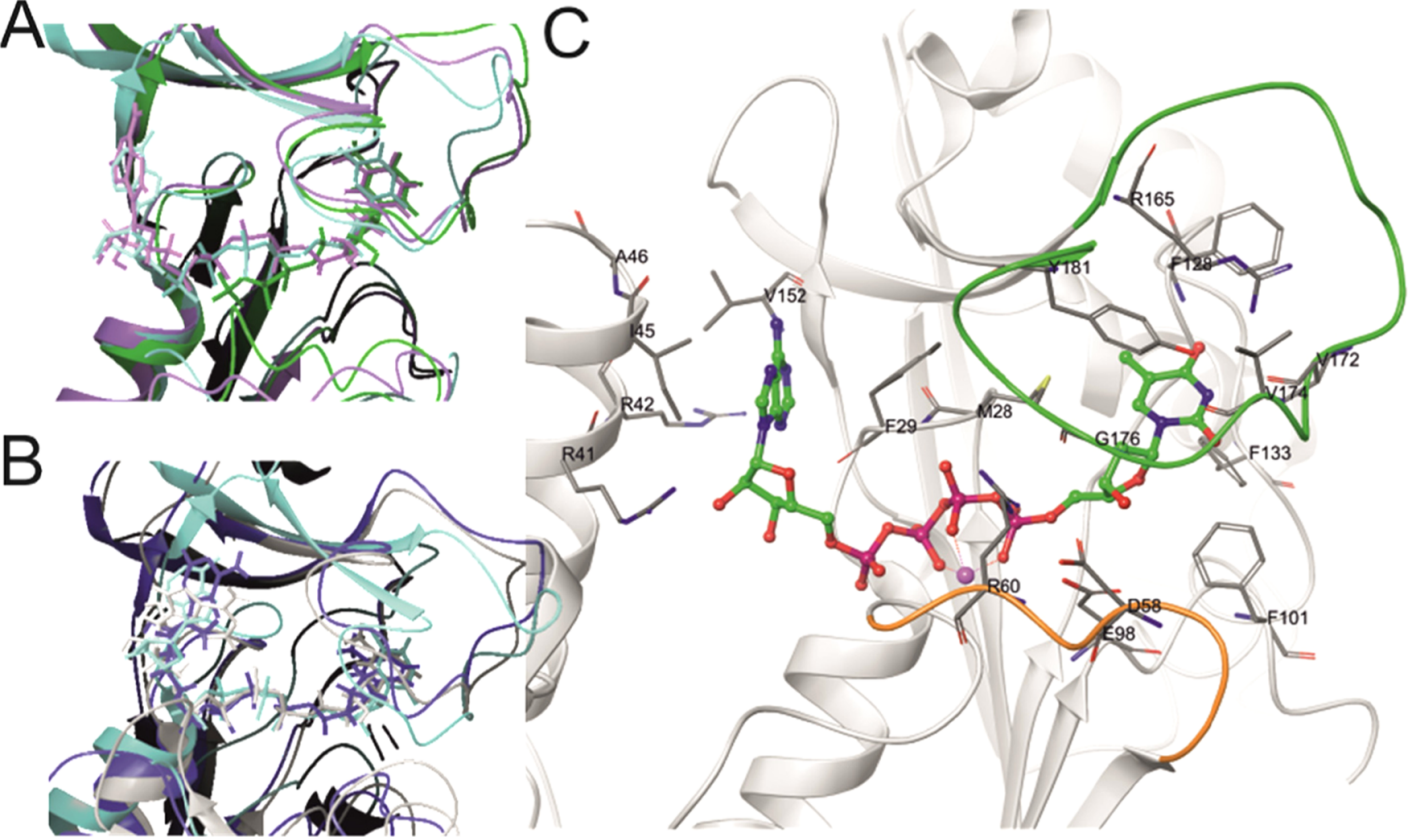
(A,B) Representative structure (first replica and chain A only) binding site overlaps with the 2ORW crystal structure (chain B, cyan): hTK1 structures (A, pink: hTK1-4TA and green: hTK1-TTP) and *Tm*TK control systems (B, gray: *Tm*TK-DIM and blue: *Tm*TK-TET). (C) Stereo view of the active center of the hTK1-4TA representative (chain A) with the flexible loops discussed in the text marked orange (amino acids no. 56–61) and green (166–180).

As expected, the adenosine in 4TA, which was introduced into hTK1 to induce the conformational changes leading to an open conformation, occurs in *syn* conformation and interacts with the neighboring monomer in all simulations. On average, one hydrogen bond is formed between the adenosine in 4TA and the neighboring protein chain for hTK1-4TA. Adenosine oxygen atoms interact with Arg41 of the neighboring monomer for more than 50% of simulation time for most monomers. Additionally, Arg42, Ile45, and Ala46 form hydrogen bonds for up to 10% of simulation time each (Figure 5C). In the experimental studies performed for *Tm*TK, two hydrogen bonds are reported between the adenosine moiety and a neighboring subunit.^12^ Interestingly, without these interactions, the adenosine moiety assumes a different conformation from that in the crystal structure, as indicated by simulations for *Tm*TK-DIM (see Figure 5A,B for the overlap of the representative ligand-binding sites, the ligand dihedral angle analysis is shown in Figure S4). Other stabilizing interactions for hTK1-4TA are the interactions with Val152 and Phe29 (of the same subunit as the ligand). These correspond to Val139 and Tyr13 described for *Tm*TK in the literature.^11^

Thymidine, being bound at a more buried site, is not expected to change over the course of simulation because all crystal structures used to prepare the simulations were bound to this substrate on that site. Indeed, the loop responsible for thymidine binding (166–180) is stable in all simulations (see root mean square fluctuation (RMSF) plots in Figure S5). This loop is more stable (up to 2 Å RMSFs) for most amino acids compared to residues 56–61 of the phosphate- and adenosine-binding site, which exhibit an RMSF of ∼3 Å, and the ligand atoms’ RMSF (Figure S6) shows major fluctuations (up to 4 Å) only at the deoxyadenosine atoms.

The thymidine moiety in the hTK1 structures is positioned in the hydrophobic pocket (Figure 5C) and is hydrogen bonded to atoms of Phe128, Val172, Val174, and Gly176 and Asp58, additionally stacked against the conserved Arg165 and Tyr181 in the lasso-domain site and to two phenylalanines (Phe133 and Phe101) on the other site.^7^ Nine atom pair distances corresponding to these interactions were measured (see Table S5, Figure 5), demonstrating distance differences of up to 1.4 ± 1.1 Å on average over the last 900 ns of both the hTK1-4TA and hTK1-TTP simulations for all residues except Asp58. The sidechain oxygens of Asp58 are placed on average 7.8 ± 0.4 Å from the O3′ atom of thymidine in TTP; however, this distance measures only 3.6 ± 0.4 Å in 4TA.

Asp58 resides in a loop responsible for phosphate binding. The 4TA-containing simulations show at least two distinct conformations in the loop (56–61), as shown in the RMSD-2D plots (Figure S7). On the other hand, these distinct conformations are not evident in any of the hTK1-TTP simulations. The dihedral φ, ψ, and χ angle measurements of all amino acids in this region suggest that Asp58 induces these different conformations (Figure S8). The sidechain of the amino acid is pushed back if 4TA is present, which is probably caused by the fact that 4TA resembles the product state. Finally, the catalytic site configuration shows that the Glu98, which is said to serve as a catalytic base, and the Met28 constraining the 5′ position in the nucleoside (see Figure 5) do not differ on average between hTK1-4TA and hTK1-TTP (up to −0.4 ± 0.2 Å, averaged for last 900 ns of each replica), and only the Arg60 responsible for stabilizing the transition state of the reaction is pushed further (∼3.2 ± 1.0 Å, Table S6), which is also probably caused by the fact that 4TA resembles more of a product state.

The average number of hydrogen bonds formed by the 4TA ligand inside its monomer for all hTK1-4TA simulations is 8.9 ± 1.1 and is mostly created by the thymidine site of the ligand. Residues that contribute to the above-mentioned hydrogen bonds are Val172, Val174, Phe128, and Asp58, which are hydrogen bonded for 40–90% of simulation time, but also Phe29 binds for more than 90% for all simulations, Lys32 (>70%) and Gly21, Thr34 (>50%). In the literature, a total of eight hydrogen bonds is reported between 4TA and *Tm*TK: two hydrogen bonds are present within the adenosine site and six in the thymidine site.^11^ The phosphate groups of 4TA are bound to the P-loop of the subunit: the first phosphoryl group binds to Ser64 and the fourth phosphoryl group is bound to Ser62 and Arg60, and additionally, Ser30 and Lys32 are found to contribute to the binding energy, as shown by GBSA analysis. Finally, the phosphate position is also stabilized by the water network and the magnesium ion. The ion is liganded by two phosphate groups, three water molecules, and Ser33.

The approximate free energy of binding of the 4TA and TTP ligands to the protein was calculated with MM-GB(*PB*)SA^22^ (Table S7), indicating that both 4TA and TTP ligands bind with a high affinity to hTK1. Values of −186.5 ± 10.8 (GB) and −134.2 ± 12.2 (PB) kcal/mol were found for 4TA and −216.9 ± 10.5 (GB) and −230.8 ± 32.7 (PB) kcal/mol for TTP if the standard calculation protocol is used (with the internal dielectric constant equal to 1 and neglecting entropy effects). Thus, the interaction energy for TTP was larger than that for 4TA, which is easy to understand analyzing the structures of both species: 4TA has a charge of ^3–^ and is also more rigid (as bounded by the adenosine), while the charge of TTP is ^4–^ and the phosphates are free to interact with different parts of the protein. Indeed, the value of total binding energy was found to be highly affected by the internal (solute) dielectric constant (Figure S9). Studies suggest that values up to 20 could be used depending on the residues in protein.^23^ We partially addressed this problem by testing various (ε = 2–10) values of internal dielectric constant for one of the ligands in the MM-PBSA model (Figure S9) and found out that the binding energy is highly dependent on it, yet always favorable, being still equal to −58.2 kcal/mol for the highest applied constant value of 10. The contribution to the energy is mostly electrostatic, although varying from 80 to 70% of the total energy for dielectric constant equal to 1 and 10, respectively. For both 4TA and TTP, binding is mostly favored by the electrostatic contributions. For the 4TA-ligand, majority of binding energy comes from the thymidine site of 4TA and the interaction between the phosphate groups and the enzyme P-loop.

## Conclusions

The comparison of the open and closed forms of hTK1, as shown in the hTK1-4TA and hTK1-TTP simulations, respectively, shows similar trends to the experimentally reported differences between the closed and open forms of the *Tm*TK enzyme.^11^ The overall volume of the enzyme is increasing upon binding the bisubstrate inhibitor, as the enzyme expands on the weak dimer interface. These changes are accompanied by a decrease in the number of hydrogen bonds and BSA in this dimer interface. On the contrary, as expected, the strong dimer interface does not show any dramatic changes. Also, as shown in the apo simulation and literature,^11^ the thymidine moiety does not introduce any major changes in the tetramer behavior. The main characteristics fall in the same space for both hTK1-TTP and hTK1-apo simulations. We showed that the catalytically competent (open) conformation of hTK1 can be assumed in the presence of an activating bisubstrate inhibitor. With the active form of the enzyme available, one may be able to describe computationally the details of the hTK1-catalyzed phosphate transfer mechanism, including the pathway (concerted or stepwise with a pentavalent phosphorane or a metaphosphate anion intermediate) and the barriers, as well as to predict if newly proposed thymine analogues are susceptible to enzymatically controlled phosphorylation. This structure is ready to be utilized for further QM/MM mechanistic studies, and docking of new drug candidates yet before their, usually difficult and expensive, chemical synthesis. It is difficult to overestimate the accessibility of the hTK1 molecular structure for the rational design of modified nucleosides with radiosensitizing, anticancer, or antiviral properties.

## Computational Methods.

The systems were prepared with Protein Preparation Wizard^24^ and Prime^25^ of Maestro Schrödinger^26^ as well as AmberTools19.^27^ The protonation states for the protein were chosen based on the results of propKa^28^ calculations, except the histidine in the binding pocket and cysteines around Zn atoms. The bonded model of Zn was prepared with MCPB.py^29^ script supported with Gaussian09^30^ calculations. Protein parameters were assigned according to the ff14SB^31^ force field. The 4TA protonation state was chosen to be 4TA^4–^.^32^ Similarly, for the control system with a feedback inhibitor, the TTP protonation state was chosen to be TTP^4–^. The parameters for the ligands were taken from existing DT3 (bsc1)^33^ and A3 (OL3)^34^ residues; only the RESP charges for the whole molecules (4TA^4–^ and TTP^3–^) were calculated explicitly with Gaussian09^30^ at the HF/6-31 + G(d) level of theory, to ensure the consistency between its nucleoside and phosphate parts (additional polarization functions were added to account for the charged system). The missing angle parameters for the phosphate were taken from the study by Meagher, Redman, and Carlson.^35^ The detailed description of protein preparation and charge derivation procedure can be found in SI Sections 3.1 and 3.2.

The system was placed into a truncated octahedral box of TIP3P water molecules with a 15 Å buffer around the protein.^36^ Na^+^ and Cl^–^ salt ions were added to a 150 mM concentration.^37^ The full description of protein preparation can be found in SI Sections 3.1 and 3.2. A set of energy minimizations followed by 50 ps heating from 0 to 300 K, and 150 ps constant pressure and temperature equilibration (300 K, 1 atm) were performed prior to the 1.3 μs (for main simulation) or 1 μs (for control systems) MD production runs in the GPU-accelerated PMEMD^39−41^ module of AMBER18^27^ with a timestep of 2 fs (minimization/equilibration procedure description can be found in SI Section 3.3.).

The trajectories were visualized using VMD.^17^ Analyses, including the clustering procedure^38^ (SI Section 4) and global analyses (SI section 5), were performed with the CPPTRAJ^42^ module of AMBER18.^27^ The BSA between the monomers of the most populated clusters was calculated with dr_sasa^18^ and pocket volume—with EPOCK plugin.^16^

## Supporting Information Available

Figures and tables concerning the simulation analyses, that is, tetramer expansion (radius of gyration, protein dimensions, pocket volume, and BSA along with hydrogen bonds between monomer analysis results), active site, and ligand conformation and binding mode (including RMSF and distances between the ligand and binding site amino acid analysis) as well as clustering data and expanded method description with global analyses (PDF)
Coordinate files for the newly constructed model—representative structure of the most populated cluster from the first hTK1-4TA replica with waters 5 Å around the ligand preserved (PDB)

## Supplementary Material

ci1c01157_si_001.pdf

ci1c01157_si_002.pdb

## Notes

Computational instructions and initial data for this work were described in the main text and in the Supporting Information; coordinates of the structures used to create initial models for this study were retrieved from the Protein Data Bank,^13^ and the structure of open conformation hTK1 resulting from this study, ready for further use, is attached in a PDB format as a Supporting Information file. The trajectories and input files can be found on zenodo.org repository: 10.5281/zenodo.5772140. Further information can be obtained from S.M. (samanta.makurat@ug.edu.pl), the corresponding author. Software and codes used are as follows: (1—for system preparations) PreparationWizard^24^ and Prime^25^ of Maestro Schrödinger,^26^ MCPB.py scripts,^29^ propKa,^28^ Gaussian09,^30^ Screening Layer Tally by Container Average Potential (SLTCAP),^37^ AmberTools19 programs^27^ including tLeap; (2—for MD simulations) PMEMD^39−41^ module of AMBER18;^27^ (3—for analyses) CPPTRAJ,^42^ with its clustering procedure,^38^ MMPBSA.py,^22^ dr_sasa,^18^ VMD^17^ with EPOCK,^16^ and Normal Mode Wizard^43^ plugins. Most of the tools are freely available, and in case commercial software was used, all data to reproduce the calculations are available in the SI.

## Abbreviations

hTK1: human thymidine kinase 1
*Tm*TK: *Thermotoga maritima* TK
ATP: adenine triphosphate
T: thymidine
TTP: thymidine triphosphate
PDB: Protein Data Bank
TMP: thymidine monophosphate
4TA: P1-(5′-adenosyl)P4-(5′-(2′-deoxy-thymidyl))tetraphosphate
MD: molecular dynamics
BSA: buried surface area
PCA: principal component analysis
RMSF: root mean square fluctuations
SLTCAP: screening layer tally by container average potential
MM-PB(*GB*)SA: the molecular mechanics energies with the Poisson–Boltzmann or generalized Born and surface area continuum solvation
