# Supplementary material for: Inactive-to-Active Transition of Human Thymidine Kinase 1 Revealed by Molecular Dynamics Simulations
Source: J Chem Inf Model. 2021 Dec 17;62(1):142–9. doi: 10.1021/acs.jcim.1c01157 (PMC8757434; doi:10.1021/acs.jcim.1c01157)
Supplement: Supplementary file 1 — ci1c01157_si_001.pdf [file ci1c01157_si_001.pdf]

SUPPORTING INFORMATION

Inactive to active transition of human Thymidine Kinase 1 revealed by Molecular Dynamics simulations

Samanta Makurat[*1], *Zoe Cournia*[2], *Janusz Rak*[1]

[1] Faculty of Chemistry University of Gdańsk, Wita Stwosza 63, 80-308 Gdańsk, Poland

[2] Biomedical Research Foundation, Academy of Athens, 4 Soranou Ephessiou, 11527 Athens, Greece

Corresponding Author

* e-mail: samanta.makurat@ug.edu.pl

1. Tetramer expansion ... 3
1.1. Radius of gyration ... 3
1.2. Protein dimensions ... 3
1.3. Pocket volume and buried surface area ... 4
1.4. Hydrogen bonds between monomers ... 6
2. Active site and ligand analyses ... 7
2.1. Ligand conformation ... 7
2.2. Root mean square fluctuations ... 8
2.3. Binding sites ... 10
3. Methods ... 15
3.1. Protein Preparation ... 15
3.2. Simulation preparation – AmberTools ... 16
3.3. Minimizations and MD runs ... 21
4. Clustering ... 22
5. Global analyses ... 23
REFERENCES ... 25

1. Tetramer expansion

1.1. Radius of gyration

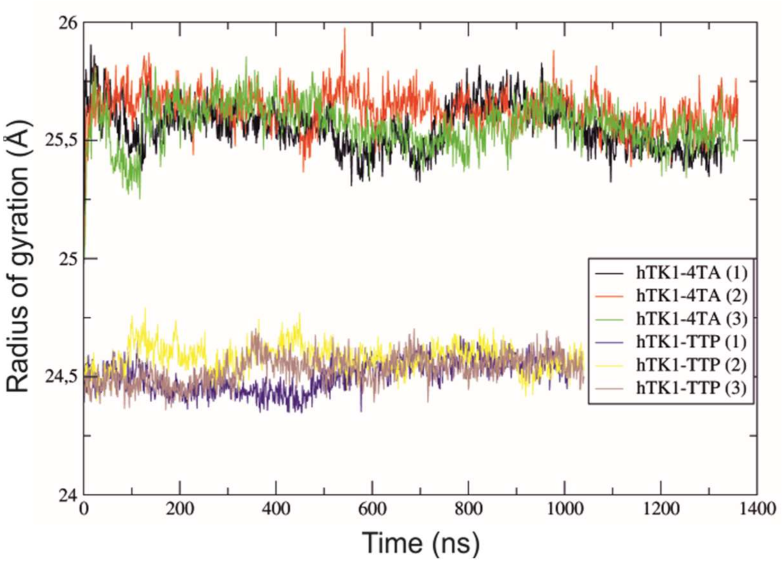

Figure S1. Radius of gyration for the Cα atoms analysis for hTK1 simulations. The replica number is shown in parentheses (1–3). The average values for radius of gyration are 25.6 ± 0.1 Å, 25.6 ± 0.1 Å and 25.6 ± 0.1 Å for hTK1-4TA replicas (1–3) and 24.5 ± 0.1 Å, 24.6 ± 0.1 Å and 24.5 ± 0.1 Å for hTK1-TTP replicas (1–3). For hTK1-apo simulation, the average radius of gyration is 24.6 ± 0.1 Å.

1.2. Protein dimensions

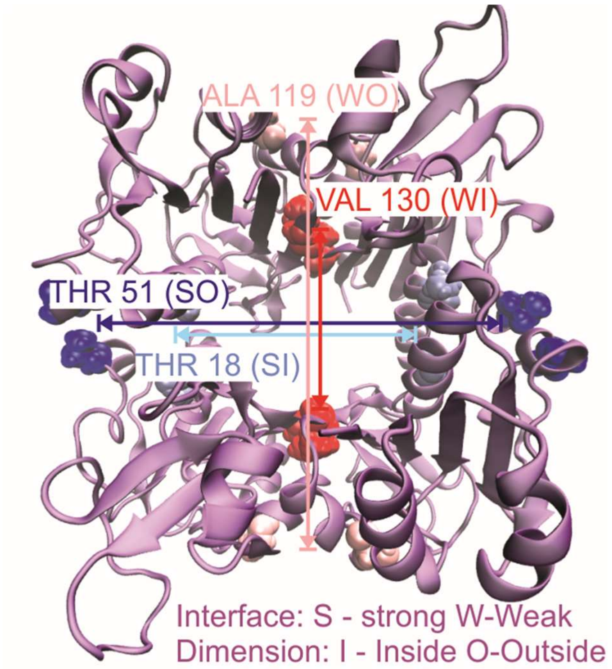

Figure S2. The aminoacids evaluated for hTK1-4TA and hTK1-TTP systems to assess the protein dimension expansion on both weak and strong dimer interfaces. The measurement was done both in the inside and the outside of the protein tetramer weak and strong interfaces, defining 4 dimensions: weak inside (WI), weak outside (WO), strong inside (SI) and strong outside (SO).

Table S1. The distances (Å) between the aminoacids describing the dimers expansion (cf. Figure S2) in the hTK1-4TA and hTK1-TTP simulations, (A) averaged over all replicas, last 900 ns and (B) for every replica separately. For hTK1-apo simulations the distances are 20.8 ± 0.5 for WI, 48.7 ± 1.0 for WO, 32.2 ± 0.5 for SI and 50.5 ± 6.5 for SO.

A

Interface	hTK1-4TA	hTK1-TTP	Difference
WI	24.2 ± 1.9	19.6 ± 0.5	4.6 ± 1.0
WO	50.2 ± 2.4	47.7 ± 0.6	2.5 ± 2.4
SI	31.0 ± 0.8	31.6 ± 0.4	-0.6 ± 0.7
SO	50.9 ± 4.9	50.1 ± 6.2	0.8 ± 8.1

B	hTK1-4TA			hTK1-TTP		
Interface	(1)	(2)	(3)	(1)	(2)	(3)
WI	23.7 ± 0.5	25.1 ± 0.7	23.6 ± 0.6	19.5 ± 0.4	19.8 ± 0.5	19.6 ± 0.6
WO	50.3 ± 2.3	50.5 ± 2.6	50.5 ± 1.8	47.5 ± 0.7	47.9 ± 0.5	47.7 ± 0.5
SI	31.5 ± 0.35	30.1 ± 0.4	31.6 ± 0.3	31.9 ± 0.3	31.3 ± 0.3	31.5 ± 0.4
SO	51.3 ± 5.1	51.7 ± 4.2	51.0 ± 5.3	50.9 ± 5.6	50.0 ± 6.2	49.3 ± 6.6

1.3. Pocket volume and buried surface area

Table S2. The binding pocket volume time average for both hTK1 simulations.

Replica	Chain	Average cavity volume (Å^3)	
		hTK1-4TA	hTK1-TTP
1	A	814.9 ± 124.6	443.4 ± 63.6
	B	701.0 ± 73.5	448.0 ± 70.9
	C	771.9 ± 63.1	409.7 ± 55.0
	D	718.9 ± 59.7	413.3 ± 61.5
2	A	925.7 ± 194.5	494.3 ± 76.3
	B	730.4 ± 110.9	425.4 ± 58.5
	C	838.5 ± 130.7	440.5 ± 74.6
	D	830.7 ± 103.6	452.0 ± 49.8
3	A	835.3 ± 131.0	405.6 ± 58.0
	B	680.1 ± 54.1	456.4 ± 106.6
	C	704.3 ± 67.4	419.9 ± 49.5
	D	836.1 ± 64.4	406.8 ± 77.8

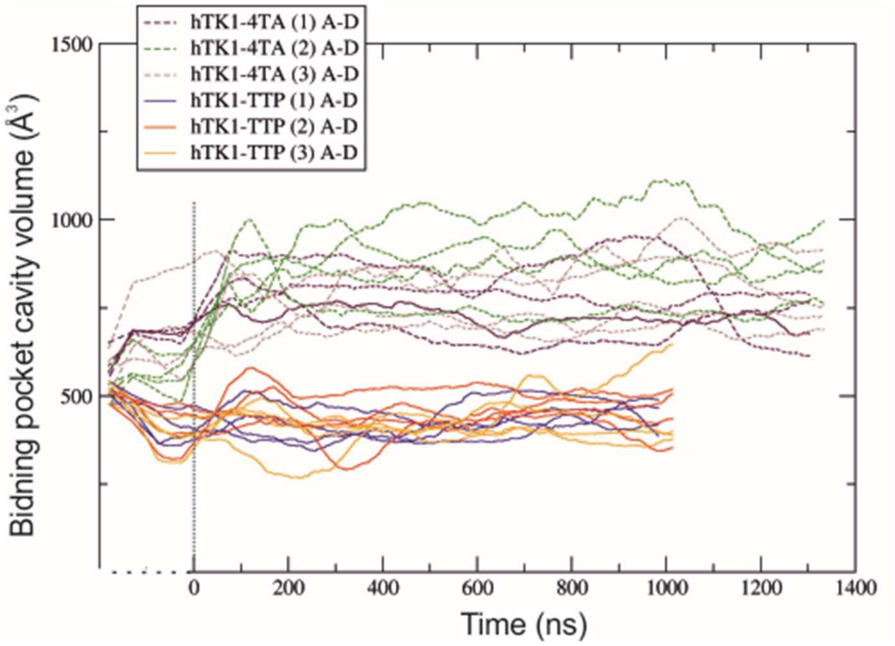

Figure S3. Running average plot of pocket volume analysis over time for both hTK1 simulations. The starting structure point, heating and equilibration points are shown (<0 ns time) to show the initial changes that occur at the very beginning of the procedure.

Table S3. The buried surface areas (BSA, Å^2) between the monomers of the most populated clusters, as calculated with dr_sasa[1].

	hTK1-4TA				hTK1-TTP			
	(1)	(2)	(3)	Average	(1)	(2)	(3)	Average
Weak interface	509.3	565.7	493.2	510.6 ± 39.1	1 146.0	1 008.0	1 137.6	1 086.9 ± 78.1
	447.8	528.0	519.8		1 183.6	1 003.6	1 042.7	
Strong interface	1 094.2	1 120.5	1 327.3	1 184.0 ± 84.2	1 182.2	1 213.9	1 169.2	1 201.8 ± 42.8
	1 216.5	1 145.5	1 200.1		1 258.2	1 239.7	1 147.7	
	TmTK-DIM				TmTK-TET			
	(1)	(2)	(3)	Average	(1)	(2)	(3)	Average
Weak interface	-				652.6	416.9	843.1	573.7 ± 179.5
					672.7	375.5	481.2	
Strong interface	1 120.6	1 204.0	1 120.6	1 148.4 ± 48.1	1 116.1	1 122.6	1 141.6	1 125.0 ± 33.0
					1 124.1	1 072.4	1 173.1	

1.4. Hydrogen bonds between monomers

Table S4. Number of hydrogen bonds on the interfaces in the simulated structures, (A) averaged for the last 900 ns of all replicas for each simulation. A default criteria of 3.0 Å distance and 135° angle cutoff were used and (B) for every simulation and every replica chain pair separately for both hTK1 simulations.

A				
	hTK1-4TA	hTK1-TTP	TmTK-DIM	TmTK-TET
Weak interface	4.0 ± 1.2	8.3 ± 1.8	-	3.5 ± 1.3
Strong interface	17.5 ± 2.1	16.9 ± 2.0	11.8 ± 2.6	11.3 ± 2.4

B	hTK1-4TA			hTK1-TTP		
Interface	(1)	(2)	(3)	(1)	(2)	(3)
Weak interface	4.9 ± 1.7	2.5 ± 1.6	4.8 ± 1.5	8.1 ± 2.3	7.8 ± 2.2	7.9 ± 2.5
	3.6 ± 1.4	4.2 ± 1.6	4.0 ± 1.5	9.6 ± 2.6	7.9 ± 2.3	8.6 ± 2.7
Strong interface	17.8 ± 2.1	17.3 ± 2.6	17.5 ± 2.3	16.8 ± 2.1	16.8 ± 2.1	17.0 ± 2.3
	17.6 ± 2.3	16.8 ± 2.6	17.6 ± 2.2	17.3 ± 2.2	17.0 ± 2.2	16.5 ± 2.4

2. Active site and ligand analyses

2.1. Ligand conformation

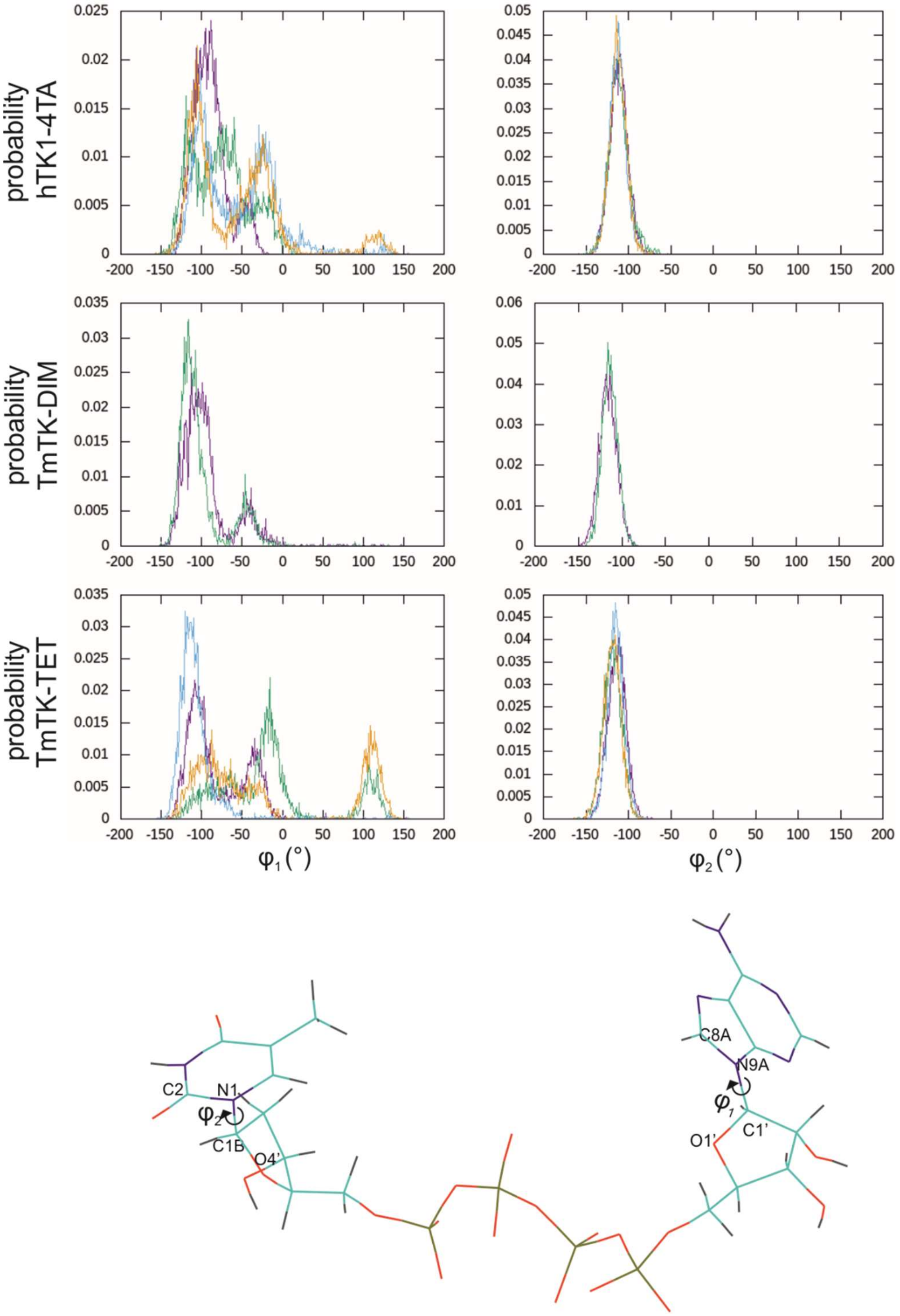

Figure S4. The dihedral angles distribution in the 4TA for hTK1-4TA, TmTK-DIM and TmTK-TET. It is visible that if a weak dimer interface is available (hTK1-4TA, TmTK-TET), the adenosine moiety explores the space differently.

2.2. Root mean square fluctuations

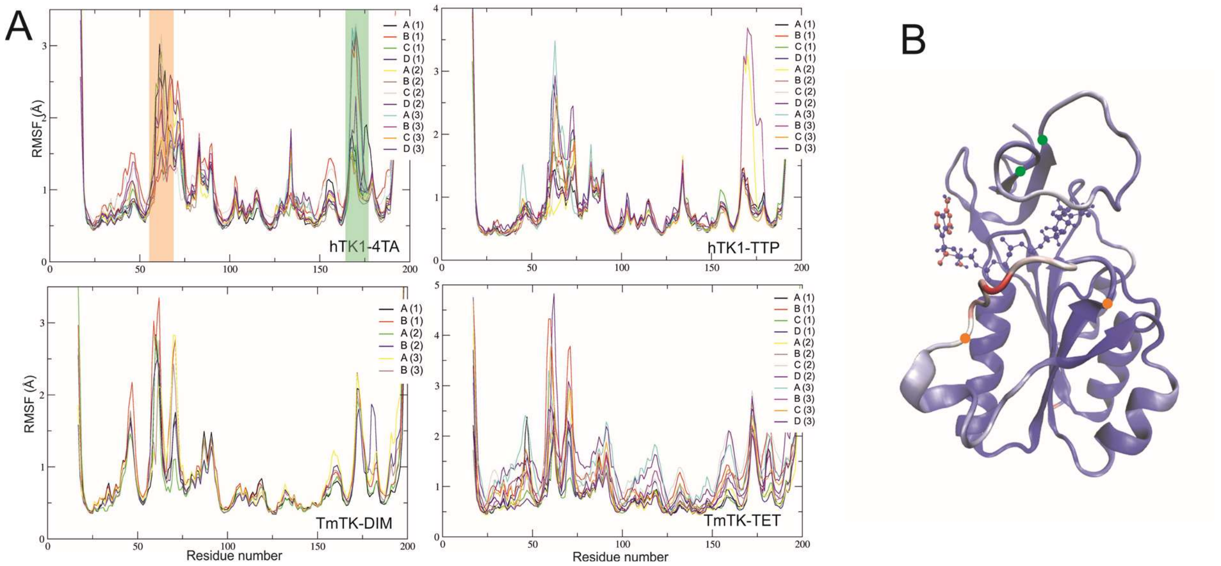

Figure S5. (A) Root mean square fluctuations (RMSF, by residue) of C, Cα and N atoms for every chain of the protein The most flexible parts are highlighted in orange (56-61) and green (166-180) for hTK1-4TA and correspond to the phosphates and thymidine binding sites, respectively. (B) Chain A, replica 1: the residues are colored by RMSF value, with blue – lowest and red – highest RMSF value. The starting and ending aminoacids for the regions of phosphates and thymidine binding sites are also marked with orange and green dots at the structure.

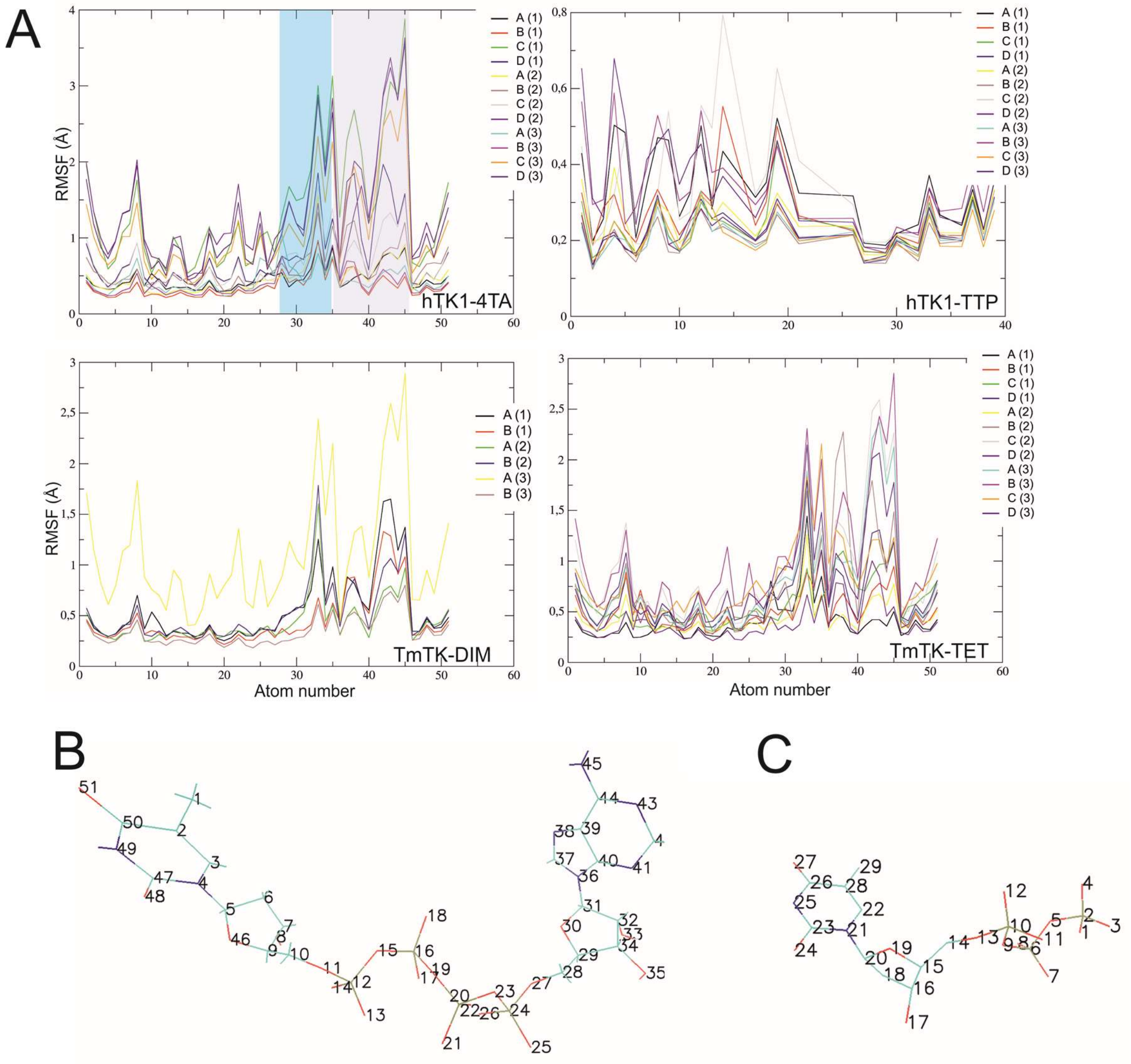

Figure S6. (A) Ligand atom RMSF (excluding hydrogen atoms). The atoms that form deoxyadenosine moiety in 4TA are marked blue (sugar, atoms no. 28-35) and violet (nucleobase, atoms no. 36-45). Ligand atoms numbering is also shown for (B) 4TA and (C) TTP.

2.3. Binding sites

Table S5. Distances (Å) between atoms described as thymidine binding site (Wellin at al.)[2] for all hTK1 simulations, averaged over last 900 ns of all replicas.

	hTK1-4TA	hTK1-TTP	Difference
Phe128@N:4TA/TTP@O4	4.4 ± 1.1	2.9 ± 0.1	1.4 ± 1.1
Val172@O:4TA/TTP@N3	3.5 ± 0.6	2.9 ± 0.2	0.6 ± 0.7
Val174@N:4TA/TTP@O2	3.8 ± 0.6	3.1 ± 0.3	0.7 ± 0.7
Gly176@N:4TA/TTP@O2	6.2 ± 0.3	6.0 ± 0.1	0.1 ± 0.3
Arg165@CA:4TA/TTP@O4	7.4 ± 0.3	7.4 ± 0.2	-0.0 ± 0.3
Tyr181@OH:4TA/TTP@O4	4.3 ± 0.2	4.4 ± 0.2	-0.1 ± 0.3
Phe133@CZ:4TA/TTP@N1	5.7 ± 0.9	5.3 ± 0.3	0.4 ± 0.9
Phe101@CZ:4TA/TTP@N1	4.8 ± 0.3	4.3 ± 0.2	0.5 ± 0.4
Asp58@OD1:4TA/TTP@O3B	3.6 ± 0.4	7.8 ± 0.4	-4.2 ± 0.5

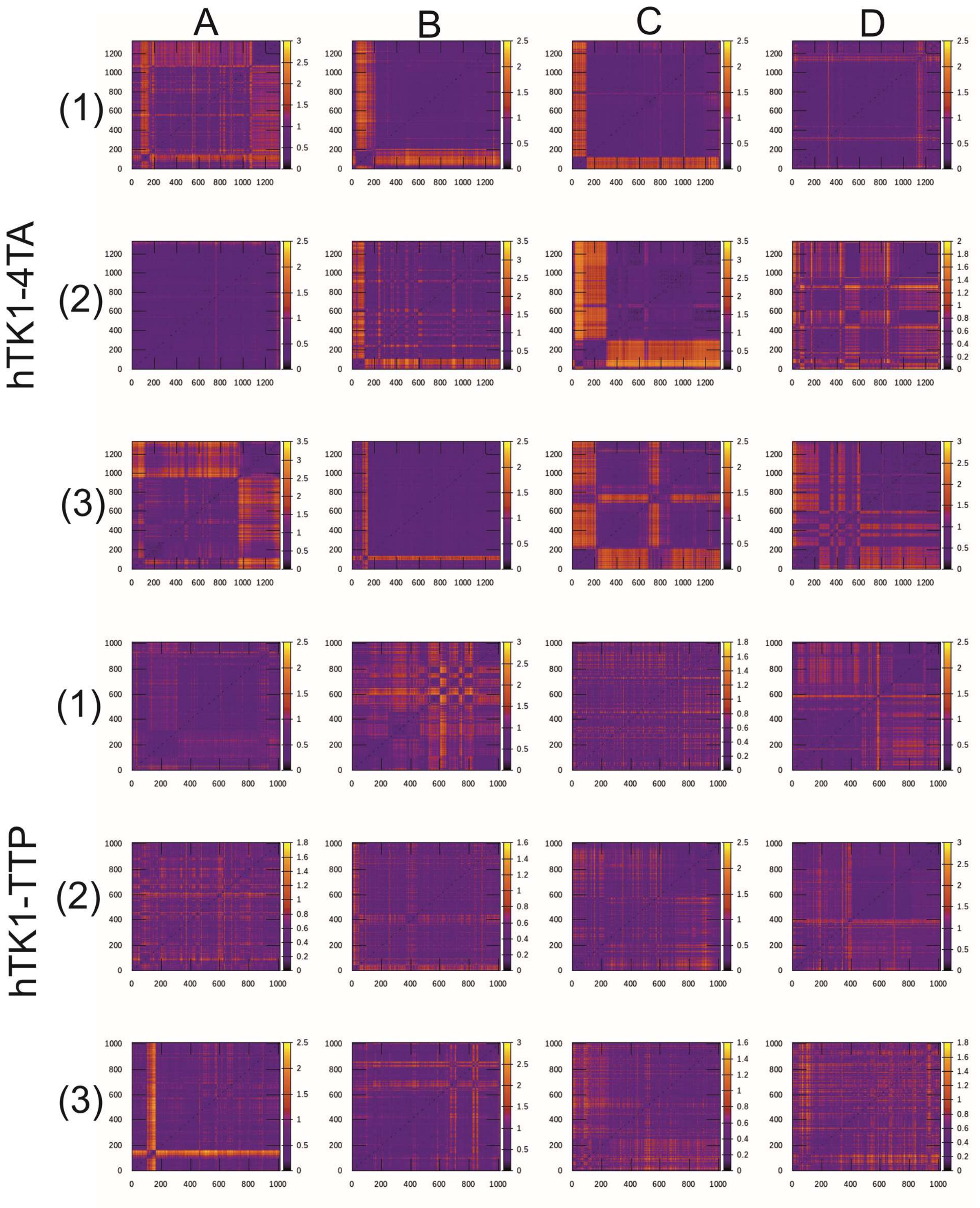
A
B
C
D
hTK1-4TA
(1)
(2)
(3)
hTK1-TTP
(1)
(2)
(3)

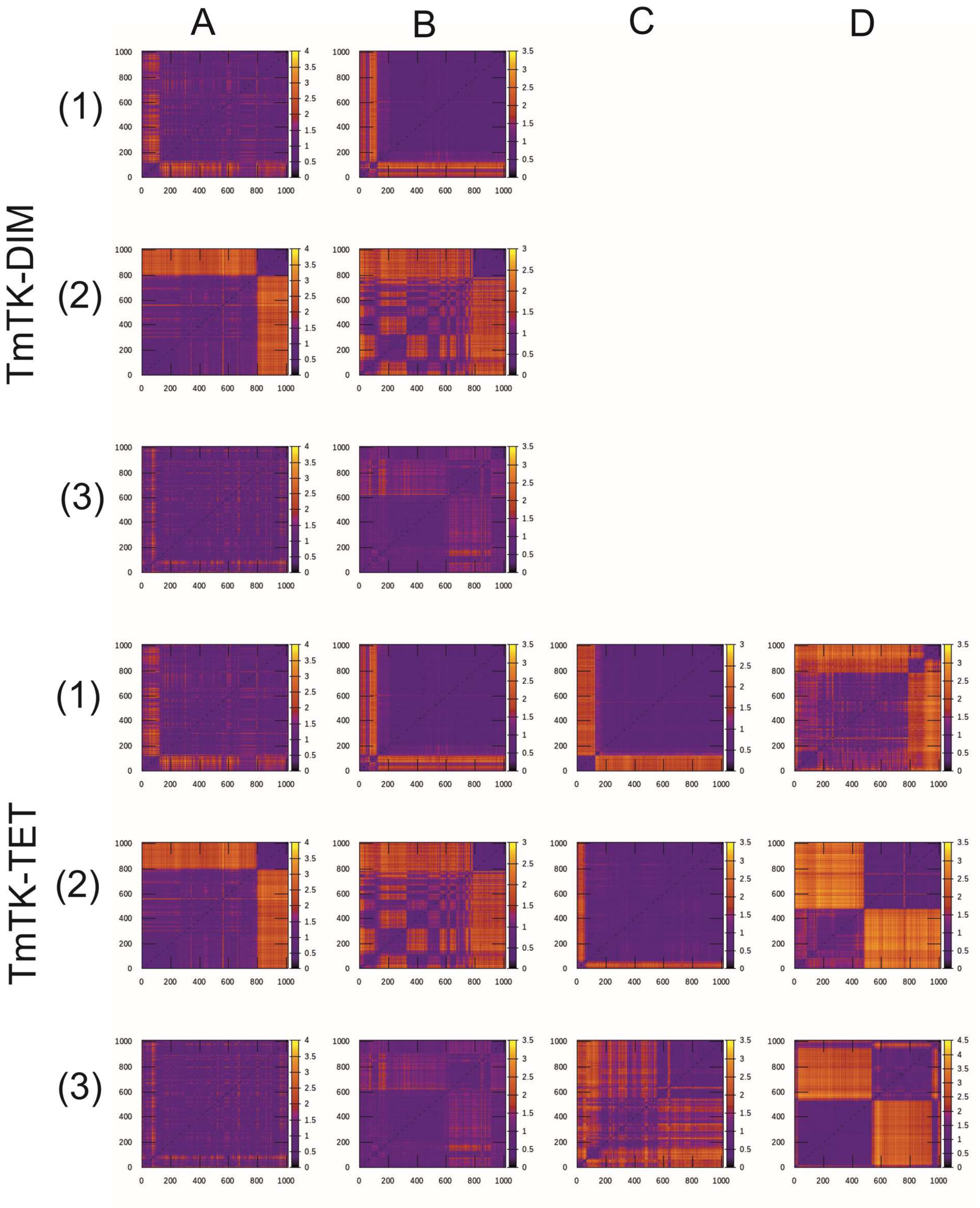

Figure S7. RMSD-2D (Å) time series for the phosphates binding (56-61 aminoacid) loop for all chains all simulations. Two distinct conformations are present for most chains of 4TA-containing simulations. Lowest RMSD – violet, highest – orange.

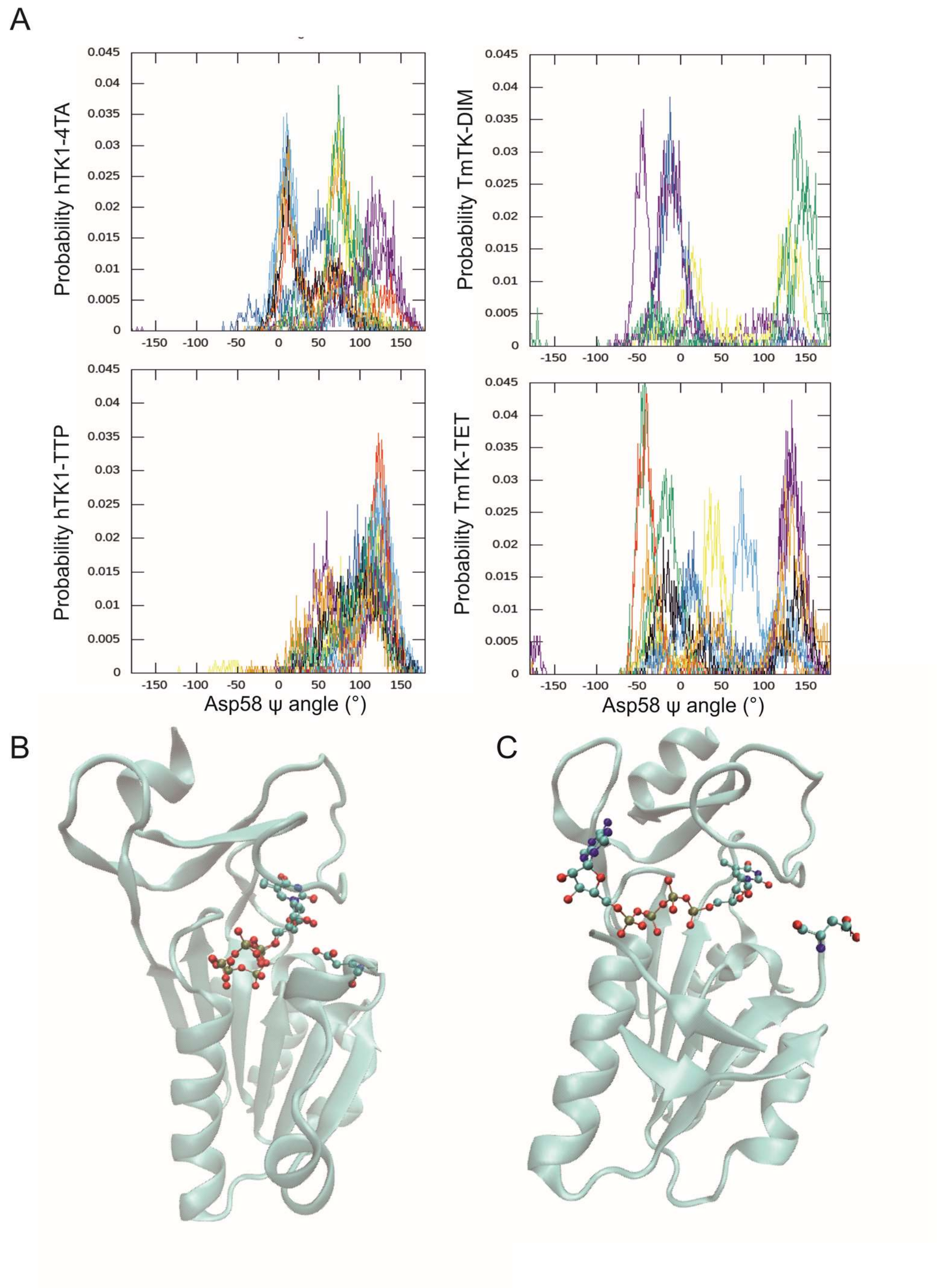

Figure S8. (A) The ψ angle representation for Asp58 in each structure. The aminoacid and the ligand are shown in ball and stick representation in the crystal structures of chain A of 1W4R (B, chain A, *h*TK1 complexed with TTP) and 2ORW (C, chain B, *Tm*TK complexed with 4TA). In the crystal structure, the neighboring aminoacid is not present, but the angle of Asp58: N, Cα, C, O is equal to -88.8° for TTP-containing structure (B) and 144.2° for 4TA-containing structure and it is visibly pushed back (C). Therefore, if the crystal structures are considered, in the first one the psi angle would be equal to 92.2°, while for the second one the angle would be -35.8°. That supports the conclusion that the Asp58 angle conversion is an answer to the substrate/product state of the ligand also in the experimental studies.

Table S6. The active-site aminoacids distances (Å^2) to C5’ atom in hTK1 simulations, averaged over last 900ns of each replica.

	hTK1-4TA	hTK1-TTP	Difference
Arg60@CZ:4TA/ATP@O5'	8.3 ± 1.1	5.1 ± 0.3	3.2 ± 1.0
Met28@SD:4TA/ATP@O5'	4.8 ± 0.2	5.2 ± 0.2	-0.4 ± 0.2
Glu98@CD:4TA/ATP@O5'	3.8 ± 0.2	3.7 ± 0.2	0.1 ± 0.3

Table S7. MM-PB(GB)SA binding energy (kcal/mol) for hTK1 replicas, averaged over all ligands. For hTK1-4TA 1 microsecond (a snapshot for every 20 ns; 51 snapshots), and for hTK1-TTP, and for TTP, 700 ns (36 frames) were analyzed.

Simulation (Replica)	MM-GBSA	MM-PBSA
hTK1-4TA(1)	-180.4 ± 12.6	-134.2 ± 12.2
hTK1-4TA(2)	-164.9 ± 11.6	-101.9 ± 16.4
hTK1-4TA (3)	-186.5 ± 10.8	-129.7 ± 11.6
hTK1-TTP (1)	-216.9 ± 10.5	-230.8 ± 32.7
hTK1-TTP (2)	-213.7 ± 11.0	-218.5 ± 15.0
hTK1-TTP (3)	-199.9 ± 14.6	-227.3 ±20.6

Figure S9. MM-PBSA binding energy values dependency on internal dielectric constant for ligand D, replica 1 of hTK1-4TA simulation. The total energy comprises several contributions, including vdW interactions, electrostatic energy as calculated by the MM force field (EEL). the electrostatic contribution to the solvation free energy calculated by PB (EPB), nonpolar (ENP) and dispersion (EDP) contributions to the solvation free energy calculated by an empirical model.

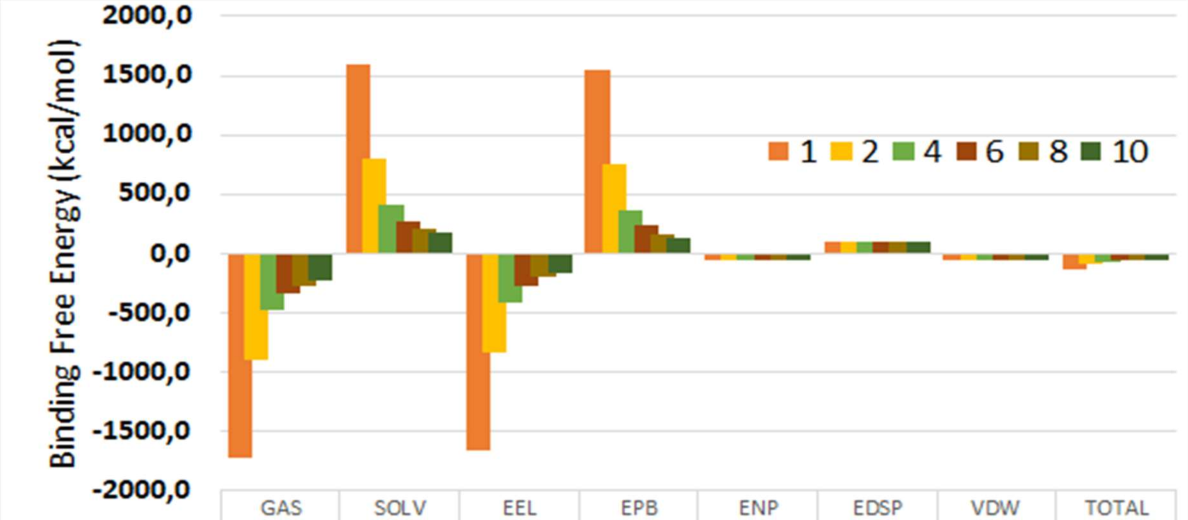

	GAS	SOLV	EEL	EPB	ENP	EDSP	VDW	TOTAL
1	-1728,0	1596,9	-1664,6	1552,9	-54,3	98,3	-63,5	-131,2
2	-895,8	799,7	-832,3	755,7	-54,3	98,3	-63,5	-96,0
4	-479,6	405,4	-416,1	361,4	-54,3	98,3	-63,5	-74,2
6	-340,9	275,0	-277,4	231,0	-54,3	98,3	-63,5	-65,9
8	-271,6	210,2	-208,1	166,2	-54,3	98,3	-63,5	-61,3
10	-229,9	171,7	-166,5	127,7	-54,3	98,3	-63,5	-58,2

3. Methods

3.1. Protein Preparation

From all available crystal structures for *h*TK1 only subunit A of 1W4R[3] contains full amino acid sequence for amino acids no. 18-191 (all other subunits lack the residues in the flexible loop in close vicinity to the catalytic center) and therefore for all the *h*TK1 simulations, the protein chain was created by four copies of 1W4R subunit A aligned to chains E-H of 1XBT[2]. This alignment allows us for proper homotetramer construction, and also for transferring of the Mg^{2+} cofactor of TTP from 1XBT to the right position since 1W4R lacks it. The amino acids of N- and C-terminal ends, <18 and >191, that are thought to possess regulatory role only, were not crystallized and are not modelled in this study. The protein was prepared with the Protein Preparation Wizard[4] of Maestro (Schrödinger)[5] (including propKa[6] at physiological pH 7.4, and termini capping, deleting water molecules further than 4 Å from the ligand). These steps prepared the core of the first control simulation of *h*TK1 that should stay in a closed state during MD (hTK1-TTP).

A few additional steps were performed constructing the hTK1-4TA simulation. Namely, all the subunits of hTK1-TTP were overlapped with chain B of 2ORW[2] in order to transfer the ligand (4TA) along with its waters and Mg^{2+} cofactor in most probable arrangement. 4TA, Mg^{2+} ion, as well as waters 4 Å around these species were replaced in the hTK1-TTP structure to these from 2ORW. It is worth mentioning here, that we also tried docking 4TA in the inactive conformation of *h*TK1, but that produced very unfavorable positions and therefore we decided to overlap both proteins and simply exchange the ligand. This procedure indeed led to proper contacts of both adenosine and thymidine sites as it is described in the literature. A few sterical clashes that are formed during the procedure are believed to lead to opening the *h*TK1 conformation during the MD.

Finally, the control simulation for *Tm*TK open-conformation (TmTK-DIM) was created by treating 2ORW crystal with Protein Preparation Wizard similarly to the previous structures, additionally missing side chains and loops were modelled with (Schrödinger)[5] and the better described monomer of TmTK-DIM (2ORW) was also multiplicated to form a homotetramer for the last control simulation (TmTK-TET).

3.2. Simulation preparation – AmberTools.

The above mentioned structures were subsequently transferred to AMBER format. Most protonation states were assigned by propKa. Glu98 in both the hTK1-4TA and hTK1-TTP simulations and Glu84 in TmTK-DIM and TmTK-TET, that serves as a base accepting the proton of the 5′-OH-group of dT in the reaction (ATP + dT → ADP + TMP, see fig. 1 of the manuscript) had to be changed to amber type GLH (4TA and TTP ligands have no hydrogen in 5′-OH-group which resembles the product state) and the histidines protonation states were addressed. Additionally, cysteines (Cys153, Cys156, Cys185, Cys188 in *h*TK1 structures, Cys140, Cys143, Cys173 and Cys176 in *Tm*TK) bonded with Zn^{2+} had to be deprotonated (CYM). The bonded model of Zn^{2+} was prepared with MCPB.py[7] script supported with Gaussian09[8] calculations. Protein parameters were assigned according to ff14SB[9] force field. The whole structures were transferred to amber format by pdb4amber[10].

The 4TA ligand atom types were assigned as in DT3 and A3 nucleosides, and therefore the parameters for these were taken from bsc1[11] and OL3[12] forcefields (Figure S10). In order to remain consistent between various parts of the molecule, the charges were calculated explicitly with Gaussian09 software, HF/6-31+G(d) level of theory for the whole molecule (Table S8). 4TA protonation state was chosen to be $^{4-}$. That is based on the standard ligands of the reaction that would be ATP and dT. The pKa of $H(ATP)^{3-}$ in water is 6.47, meaning that 89% of free ATP in solution is ATP^{4-} at physiological pH 7.4.[13] This ratio is additionally enhanced by complexation with Mg^{2+}. Prior to the charge derivation a limited optimization was performed. To keep the conformation as from the crystal structure and prevent nucleobase stacking, we kept the nucleosides rings in place (by freezing C and N atoms). To assess the quality of the charges obtained, the RMSE between calculated and force-field charges was calculated, as equal to 0.04 and 0.08 for the heavy atoms in adenosine and thymidine residues respectively (excluding CH_2-O-PO_3 ends).

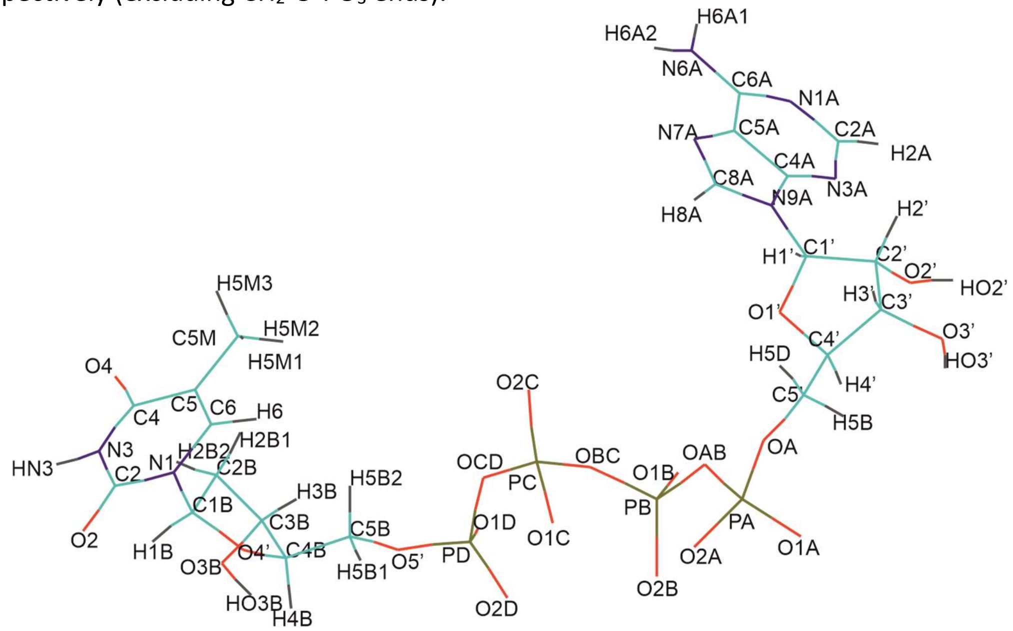

Figure S10. 4TA atom names used.

Table S8. 4TA – atom types, charges and coordinates.

No.	Atom Name	X	Y	Z	Atom Type	Charge
1	C5M	38.499	53.128	21.92	CT	-0.2214
2	C5	38.01	54.522	22.187	C2	-0.0581
3	C6	38.826	55.631	21.891	C2	-0.3165
4	N1	38.424	56.911	22.134	N*	-0.1201
5	C1B	39.237	58.064	21.73	CT	0.0567
6	C2B	40.691	57.947	22.144	CT	-0.0491
7	C3B	41.379	58.746	21.09	CE	0.404
8	O3B	41.268	60.127	21.507	OH	-0.8066
9	C4B	40.516	58.572	19.837	CT	0.0118
10	C5B	41.01	57.497	18.863	CI	0.0443
11	O5'	42.201	57.946	18.269	OS	-0.4095
12	PD	42.816	57.116	17.025	P	1.4011
13	O2D	43.329	58.166	15.994	O2	-0.9112
14	O1D	41.676	55.961	16.469	O2	-0.9112
15	OCD	43.973	56.418	17.793	OS	-0.5456
16	PC	45.491	56.356	17.427	P	1.5065
17	O1C	46.162	57.862	16.843	O2	-0.9124
18	O2C	46.15	55.356	18.434	O2	-0.9124
19	OBC	45.193	55.462	16.095	OS	-0.5863
20	PB	45.654	55.458	14.556	P	1.4609
21	O2B	45.217	56.843	13.753	O2	-0.8867
22	O1B	45.038	54.146	14.075	O2	-0.8867
23	OAB	47.251	55.216	14.525	OS	-0.5325
24	PA	48.373	56.168	13.869	P	1.4485
25	O1A	48.319	56.105	12.317	O2	-0.9334
26	O2A	48.236	57.776	14.489	O2	-0.9334
27	OA	49.684	55.511	14.451	OS	-0.5499
28	C5'	50.051	54.171	14.146	CI	0.0787
29	C4'	51.502	54.011	14.581	CT	0.1589
30	O1'	51.548	53.855	16.006	OS	-0.2745
31	C1'	52.673	53.037	16.386	CT	-0.0437
32	C2'	53.245	52.504	15.081	CT	0.681
33	O2'	54.388	53.282	14.685	OH	-0.8207
34	C3'	52.131	52.72	14.072	CT	0.2143
35	O3'	52.634	52.796	12.731	OH	-0.8399
36	N9A	52.082	52.039	17.312	N*	-0.0618
37	C8A	51.918	52.27	18.627	C5	0.1156
38	N7A	51.319	51.217	19.229	NB	-0.6398
39	C5A	51.089	50.29	18.273	CB	0.0493
40	C4A	51.583	50.831	17.009	CB	0.4269
41	N3A	51.458	50.092	15.874	NC	-0.7051
42	C2A	50.887	48.857	15.914	CQ	0.5022
43	N1A	50.405	48.312	17.043	NC	-0.8605
44	C6A	50.462	48.944	18.251	CA	0.8951

45	N6A	49.988	48.412	19.4	N2	-1.1778
46	O4'	39.236	58.13	20.285	OS	-0.4067
47	C2	37.118	57.128	22.671	C	0.7657
48	O2	36.717	58.313	22.84	O	-0.6841
49	N3	36.32	56.09	23.014	NA	-0.5387
50	C4	36.69	54.813	22.795	C	0.6727
51	O4	35.926	53.915	23.111	O	-0.6933
52	H5M1	37.721	52.523	21.455	HC	0.0708
53	H5M2	39.352	53.125	21.242	HC	0.0708
54	H5M3	38.8	52.646	22.85	HC	0.0708
55	H6	39.784	55.513	21.385	H4	0.373
56	H1B	38.828	58.98	22.158	H2	0.1851
57	H2B1	41.079	56.938	22.119	HC	0.0134
58	H2B2	40.831	58.318	23.158	HC	0.0134
59	H3B	42.42	58.46	20.939	H1	0.0594
60	HO3B	41.656	60.688	20.822	HO	0.4774
61	H4B	40.392	59.505	19.288	H1	0.1323
62	H5B1	40.25	57.346	18.095	H1	0.048
63	H5B2	41.169	56.55	19.376	H1	0.048
64	H4'	52.1	54.875	14.291	H1	0.0301
65	H1'	53.365	53.667	16.946	H2	0.0842
66	H2'	53.539	51.458	15.166	H1	-0.1192
67	HO2'	54.775	52.874	13.905	HO	0.4592
68	H3'	51.399	51.914	14.135	H1	0.0372
69	HO3'	53.017	53.669	12.591	HO	0.4983
70	H8A	52.233	53.201	19.098	H5	0.214
71	H2A	50.817	48.287	15	H5	0.0869
72	H6A1	49.583	47.476	19.388	H	0.4748
73	H6A2	50.05	48.949	20.265	H	0.4748
74	HN3	35.395	56.287	23.396	H	0.3548
75	H5D	49.408	53.464	14.67	H1	0.089
76	H5B	49.941	53.972	13.08	H1	0.089

For TTP (hTK1-TTP) the missing phosphate parameters were taken from Meagher at al[14], O3 atom type was added for the terminal phosphate. The charges were calculated similarly to the 4TA ligand charges (Gaussian09, HF/6-31+G(d), C, N and P atoms were frozen to prevent the structure from obtaining a geometry far from this from crystal structure). Atom types as well as the charges used are shown in Figure S11 and Table S9.

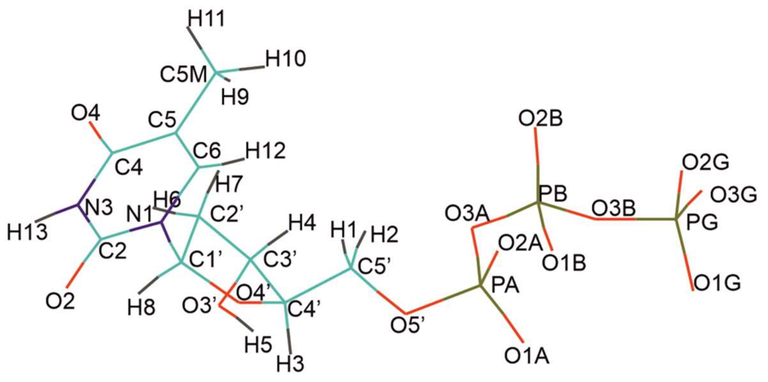

Figure S11. TTP atom names used.

Table S9. TTP – atom types, charges and coordinates.

No.	Atom Name	X	Y	Z	Atom Type	Charge
1	O2G	47.401	55.061	15.862	O3	-1.1034
2	PG	46.124	55.232	15.096	P	1.6998
3	O1G	46.074	56.406	14.16	O3	-1.1034
4	O3G	45.586	53.928	14.562	O3	-1.1034
5	O3B	45.039	55.582	16.208	OS	-0.4322
6	PB	45.392	56.254	17.629	P	1.6398
7	O1B	46.135	57.548	17.334	O2	-1.0127
8	O2B	45.952	55.195	18.531	O2	-1.0127
9	O3A	43.903	56.611	18.124	OS	-0.6013
10	PA	42.746	56.833	17.025	P	1.3713
11	O1A	43.294	57.739	15.952	O2	-0.8743
12	O2A	42.167	55.493	16.676	O2	-0.8743
13	O5'	41.608	57.631	17.843	OS	-0.6
14	C5'	41.417	57.296	19.207	CT	0.0622
15	H1	40.78597	56.41689	19.29847	H1	0.0276
16	H2	42.39242	57.03363	19.62195	H1	0.0276
17	C4'	40.784	58.432	19.984	CT	0.2958
18	C3'	41.53	58.58	21.297	CE	0.2962
19	O3'	41.641	59.965	21.623	OH	-0.802
20	H5	42.16554	60.37425	20.94767	HO	0.4415
21	C2'	40.682	57.869	22.33	CT	-0.0722
22	H6	40.7559	58.32891	23.30771	HC	0.024
23	H7	41.00477	56.83497	22.40745	HC	0.024
24	H4	42.53775	58.19584	21.23979	H1	0.0802
25	H3	40.71325	59.3462	19.41905	H1	0.0363
26	O4'	39.429	58.095	20.311	OS	-0.4407
27	C1'	39.284	57.983	21.73	CT	0.0061
28	H8	38.76767	58.84708	22.10449	H2	0.1957
29	N1	38.423	56.851	22.081	N*	0.018

30	C6	38.817	55.569	21.886	C2	-0.2974
31	H12	39.77002	55.40816	21.42224	H4	0.3552
32	C2	37.211	57.149	22.607	C	0.6729
33	O2	36.898	58.351	22.764	O	-0.7146
34	N3	36.334	56.187	22.953	NA	-0.4413
35	H13	35.45101	56.46609	23.31968	H	0.3336
36	C4	36.635	54.896	22.808	C	0.5987
37	O4	35.813	54.025	23.15	O	-0.679
38	C5	37.951	54.53	22.235	C2	-0.0561
39	C5M	38.327	53.082	22.044	CT	-0.1361
40	H9	37.61745	52.56935	21.40294	HC	0.0502
41	H10	39.30439	53.01354	21.58493	HC	0.0502
42	H11	38.35048	52.54786	22.98927	HC	0.0502

All the systems were solvated in a 15 Å octahedral box of TIP3P water away from the protein and neutralized with LEap, the number of ions required for implementing the physiological pH of 150mM was calculated according to Screening Layer Tally by Container Average Potential (SLTCAP) tool[15] (the protein mass used were 100 kDa for the tetramer and 50 kDa for the dimer[16]). The final number of atoms in each model is summarized in Table S10.

Table S10. Protein, ligand, water and ion atoms numbers in each simulation:

	hTK1-4TA	hTK1-TTP	TmTK-DIM	TmTK-TET
Protein	10984	10984	5726	11460
Ligand	304	168	152	304
Water	57459	54786	48768	58668
Mg^{2+}	4	4	4	4
Zn^{2+}	4	4	4	4
Na^{+}	38	39	43	52
Cl^{-}	42	43	34	34
Total	68835	66028	54731	70526

3.3. Minimizations and MD runs

Since the simulations (especially the main one), contain numerous overlaps with atoms in other residues, a set of steepest descent (SD) followed by conjugate gradient (CG) minimizations were run for all the systems: [1] 500 SD and 500 CG for waters and salt only, [2] 500 SD and 500 CG for protein atoms only and [3] 1500 SD and 1500 CG with no restraints. These were followed by 50 ps heating from 0 to 300 K (constant volume with a weak, 10 Å, restraint on a solute and time step of 0.5 fs) and finally – equilibration. The equilibration consisted of 50 ps with a 0.5 fs time step and 100 ps with a 1 fs time step of constant temperature [300 K] and pressure [1 atm] simulation without any restraints. Periodic boundary simulations based on the particle mesh Ewald (PME), 10 Å cutoff on nonbonded interactions and SHAKE algorithm constraining bonds involving hydrogen were used along with the Langevin thermostat to control the temperature (collision frequency of 1 ps^{-1}).

4. Clustering

The clustering procedure was performed with K-means algorithm as implemented in CPPTRAJ[17] module of AMBER18[10]. The method was chosen from the various clustering algorithms basing on the most favorable DBI, pSF and SSR/SST ratio values[18] and visual inspection of the results variation for the first hTK1-4TA simulation. 15 clusters were produced for each system. The cluster analysis applied to the whole production runs, basing on the RMSD of the backbone atoms. The centroid of each cluster was considered to be the most representative structure of the cluster. For most cases, the first and most populated cluster (0) included more than 20% of the simulation, and the second one - at least a few percentage points less (Table S11), therefore only the snapshot corresponding to the most populated cluster is discussed in the text.

Table S11 Clustering analysis results for the most populated clusters.

		cluster centroid frame (and fraction)		
Simulation	Cluster no.	Replica 1	Replica 2	Replica 3
hTK1-4TA	0	445 (23%)	816 (32%)	668 (21%)
	1	1246 (16%)	384 (20%)	1237 (16%)
hTK1-TTP	0	332 (21%)	712 (16%)	411 (20%)
	1	859 (14%)	521 (14%)	209 (15%)
TmTK-DIM	0	531 (24%)	931 (24%)	717 (36%)
	1	701 (16%)	755 (21%)	435 (17%)
TmTK-TET	0	866 (47%)	493 (32%)	557 (31%)
	1	642 (15%)	912 (17%)	957 (27%)

5. Global analyses

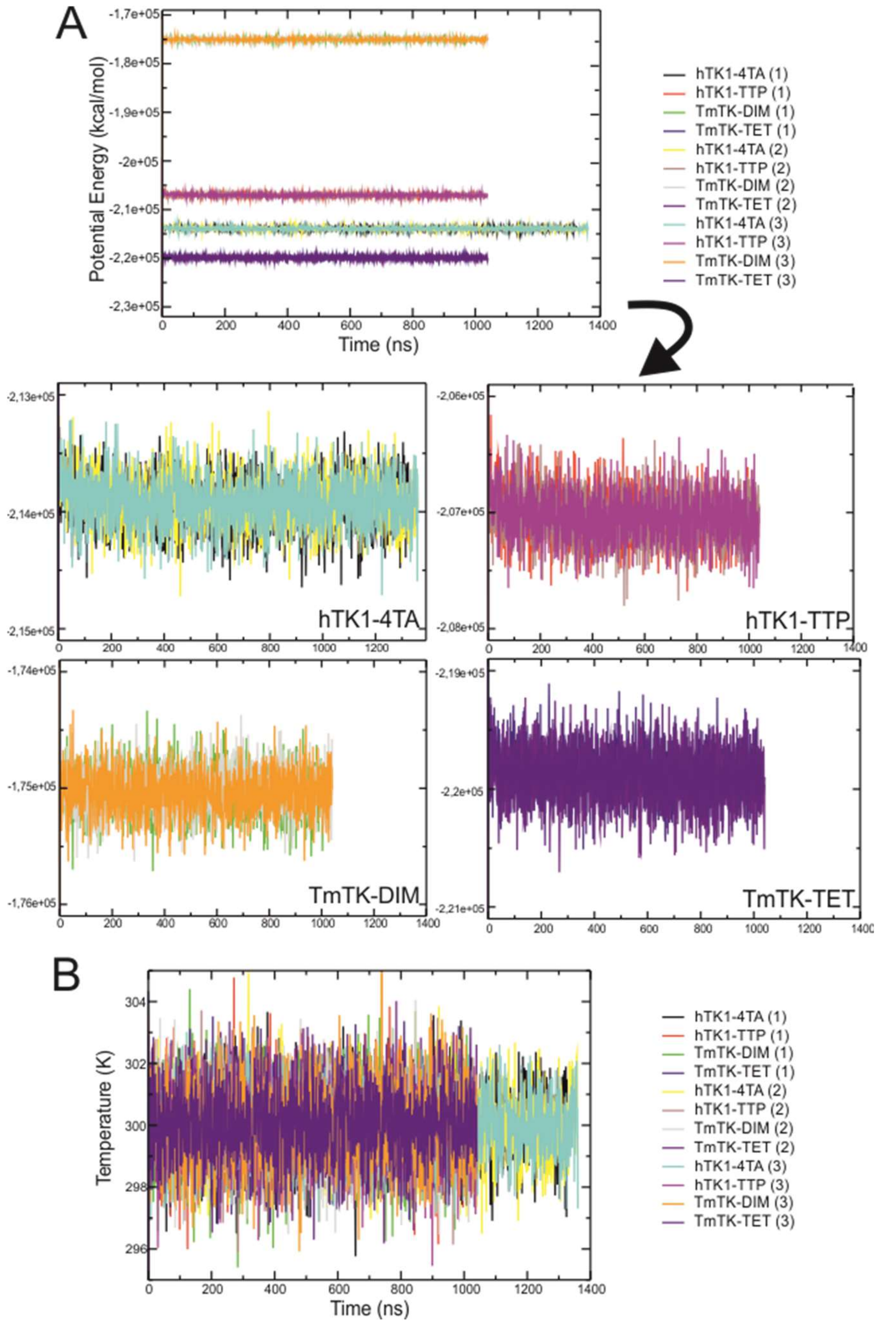

Figure S12. Time series of the total potential energy (A) and Temperature fluctuations (B) over the course of the simulation for all the systems. Number of replica (1–3) is shown in the parentheses in the legend.

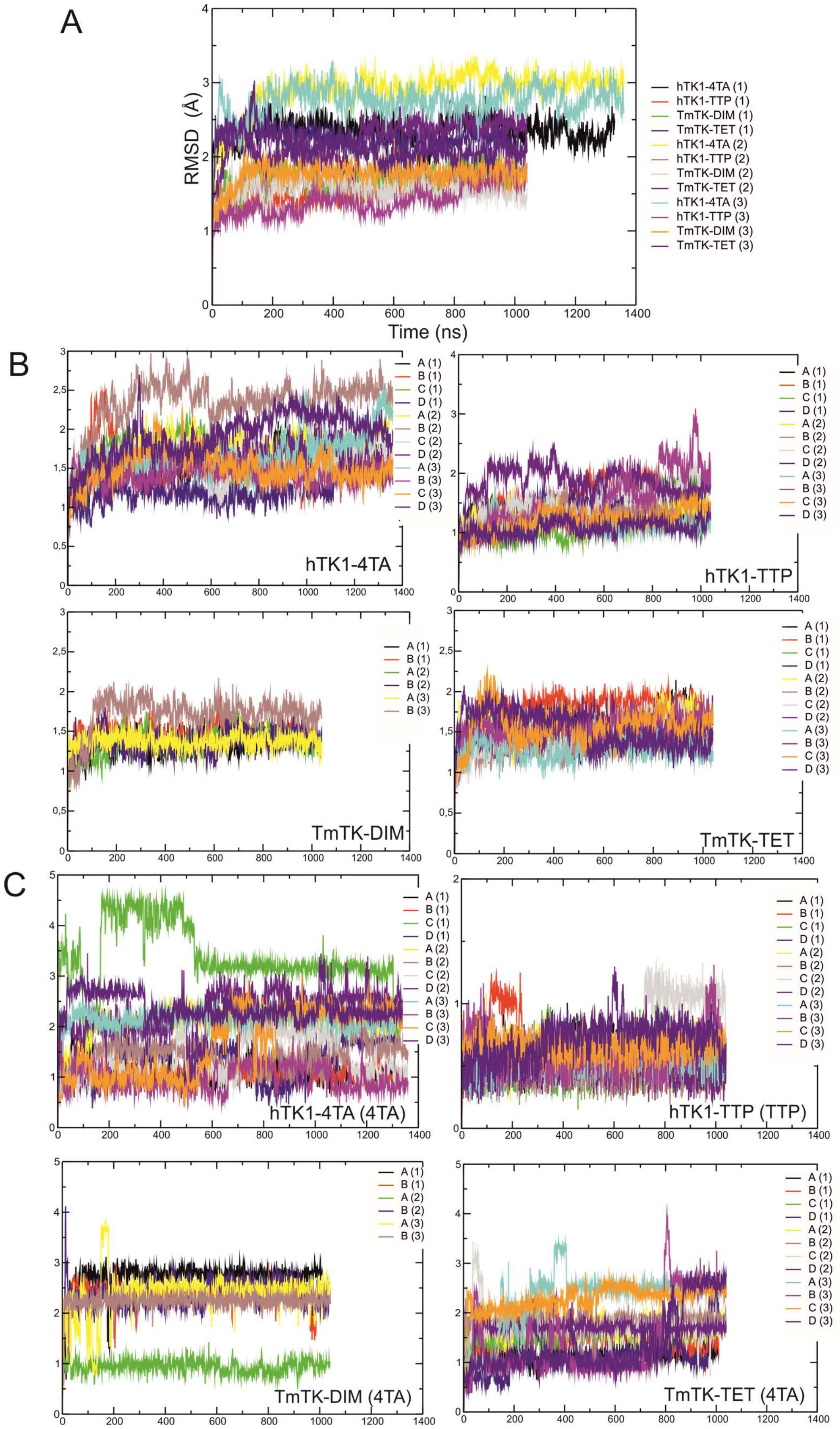

Figure S13. Time series of RMSD with the respect to the equilibrated structures for all systems (A), all chains in each system (B) and all ligands (C). Number of replica (1–3) is shown in the parentheses in the legend.

REFERENCES

1. Ribeiro, J.; Ríos-Vera, C.; Melo, F.; Schüller, A., Calculation of accurate interatomic contact surface areas for the quantitative analysis of non-bonded molecular interactions. *Bioinformatics* **2019,** *35*, 3499-3501.
2. Welin, M.; Kosinska, U.; Mikkelsen, N.-E.; Carnrot, C.; Zhu, C.; Wang, L.; Eriksson, S.; Munch-Petersen, B.; Eklund, H., Structures of thymidine kinase 1 of human and mycoplasmic origin. *Proc. Natl Acad. Sci.* **2004,** 1*0*1, 17970-17975.
3. Segura-Peña, D.; Lichter, J.; Trani, M.; Konrad, M.; Lavie, A.; Lutz, S., Quaternary structure change as a mechanism for the regulation of thymidine kinase 1-like enzymes. *Structure* **2007,** 1*5*, 1555-1566.
4. Sastry, G. M.; Adzhigirey, M.; Day, T.; Annabhimoju, R.; Sherman, W., Protein and ligand preparation: parameters, protocols, and influence on virtual screening enrichments. *J. Comput. Aided Mol. Des.* **2013,** *27*, 221-234.
5. ***Schrödinger Release 2020-2*** *: Maestro, Schrödinger, LLC, New York, NY, 202*1.
6. Olsson, M. H.; Søndergaard, C. R.; Rostkowski, M.; Jensen, J. H., PROPKA3: Consistent Treatment of Internal and Surface Residues in Empirical pKa Predictions. *J. Chem. Theory Comput.* **2011,** *7*, 525-537.
7. Li, P.; Merz, K. M., MCPB.py: A Python Based Metal Center Parameter Builder. *J. Chem. Inf. Model* **2016,** *56*, 599-604.
8. Frisch, M. J.; Trucks, G. W.; Schlegel, H. B.; Scuseria, G. E.; Robb, M. A.; Cheeseman, J. R.; Scalmani, G.; Barone, V.; Petersson, G. A.; Nakatsuji, H., *Gaussian 09*, D.01.; Gaussian, Inc.: Wallingford CT, 2009.
9. Maier, J. A.; Martinez, C.; Kasavajhala, K.; Wickstrom, L.; Hauser, K. E.; Simmerling, C., ff14SB: Improving the Accuracy of Protein Side Chain and Backbone Parameters from ff99SB. *J. Chem. Theory Comput.* **2015,** 11, 3696-3713.
10. Case, D.A.; Ben-Shalom, I. Y.; Brozell, S.R.; Cerutti, D.S.; Cheatham, T.E.; Cruzeiro, V.W.D; Darden, T.A.; Duke, R.E.; Ghoreishi, D.; Giambasu, G., *AMBER 2019*, University of California, San Francisco, 2019.
11. Ivani, I.; Dans, P. D.; Noy, A.; Pérez, A.; Faustino, I.; Hospital, A.; Walther, J.; Andrio, P.; Goñi, R.; Balaceanu, A.; Portella, G., Parmbsc1: a refined force field for DNA simulations. *Nat. Methods* **2016,** 1*3*, 55-58.
12. Zgarbová, M.; Otyepka, M.; Sponer, J.; Mládek, A.; Banáš, P.; Cheatham, T. E.; Jurečka, P., Refinement of the Cornell et al. Nucleic Acids Force Field Based on Reference Quantum Chemical Calculations of Glycosidic Torsion Profiles. *J. Chem. Theory Comput.* **2011,** *7*, 2886-2902.
13. Sigel, H.; Tribolet, R.; Malini-Balakrishnan, R.; Martin, R. B., Comparison of the stabilities of monomeric metal ion complexes formed with adenosine 5'-triphosphate (ATP) and pyrimidine-nucleoside 5'-triphosphate (CTP, UTP, TTP) and evaluation of the isomeric equilibria in the complexes of ATP and CTP. *Inorg. Chem.* **1987,** *26*, 2149-2157.
14. Meagher, K. L.; Redman, L. T.; Carlson, H. A., Development of polyphosphate parameters for use with the AMBER force field. *J. Comput. Chem.* **2003,** *24*, 1016-1025.
15. Schmit, J. D.; Kariyawasam, N. L.; Needham, V.; Smith, P. E., SLTCAP: A Simple Method for Calculating the Number of Ions Needed for MD Simulation. *J. Chem. Theory Comput.* **2018,** 1*4*, 1823-1827.

16. Munch-Petersen, B., Reversible tetramerization of human TK1 to the high catalytic efficient form is induced by pyrophosphate, in addition to tripolyphosphates, or high enzyme concentration. *FEBS J.* **2009,** *276*, 571-80.
17. Roe, D. R.; Cheatham, T. E., PTRAJ and CPPTRAJ: Software for Processing and Analysis of Molecular Dynamics Trajectory Data. *J Chem Theory Comput* **2013,** *9*, 3084-95.
18. Shao, J.; Tanner, S. W.; Thompson, N.; Cheatham, T. E., Clustering Molecular Dynamics Trajectories: 1. Characterizing the Performance of Different Clustering Algorithms. *J Chem Theory Comput* **2007,** *3*, 2312-34.
